# Geohistorical records indicate no impact of the Deepwater Horizon oil spill on oyster body size

**DOI:** 10.1098/rsos.160763

**Published:** 2016-11-30

**Authors:** Gregory P. Dietl, Stephen R. Durham

**Affiliations:** 1 Paleontological Research Institution, Ithaca, NY 14850, USA; 2 Department of Earth and Atmospheric Sciences, Cornell University, Ithaca, NY 14853, USA

**Keywords:** baseline, before-after-control-impact analysis, *Crassostrea virginica*, death assemblage, environmental assessment

## Abstract

Documentation of the near- and long-term effects of the Deepwater Horizon (DWH) oil spill, one of the largest environmental disasters in US history, is still ongoing. We used a novel before-after-control-impact analysis to test the hypothesis that average body size of intertidal populations of the eastern oyster (*Crassostrea virginica*) inhabiting impacted areas in Louisiana decreased due to increased stress/mortality related to the oil spill. Time-averaged death assemblages of oysters were used to establish a pre-spill baseline of body-size structure for four impacted and four control locations along a 350 km stretch of Louisiana's coastline. Post-spill body sizes were then measured from live oysters at each site in order to evaluate the differences in body size between oiled (i.e. impact) and unoiled (i.e. control) locations before and after the spill. Our results indicate that average body size of oysters remained relatively unchanged after the oil spill. There were also no temporal patterns in temperature, salinity or disease prevalence that could have explained our results. Together, these findings suggest that oysters either recovered rapidly following the immediate impact of the DWH oil spill, or that its impact was not severe enough to influence short-term population dynamics of the oyster beds.

## 1. Introduction

The Deepwater Horizon (DWH) oil spill began on 20 April 2010 and became one of the worst environmental disasters in US history. Over 87 days, 3.19 million barrels of oil poured into the northern Gulf of Mexico, resulting in widespread environmental and economic damage to the Gulf Coast and a massive, still-ongoing assessment and remediation effort [1]. In the years since the spill, many studies have documented significant direct and indirect effects of both the oil spill itself and the human response to it [2–8]. For instance, field surveys have documented 77% lower cover of oysters (*Crassostrea virginica*)—one of the most ecologically and economically important Gulf Coast species [9]—in near shore areas that experienced heavy oiling relative to reference locations that were not oiled [10]. Damage to oyster habitat was also related to oil clean-up and spill response activities, including estimated losses of over one billion adult-equivalent oysters, killed by emergency freshwater diversions from the Mississippi River conducted in response to the oil spill [10–13].

Such acute impacts are often assessed using comparisons with reference sites that varied in exposure to oil contamination, but this approach overlooks the possibility that impacts may exist at these sites despite a lack of visible oil. This issue highlights a common source of uncertainty in many scientific assessments of the ecological effects of oil spills—insufficient baseline information (e.g. [4,10]). Many DWH oil spill impact estimates were based on comparisons between areas that differed in the levels of observed oil, or baseline samples hastily collected before oil reached the coast [1,4]. However, there is no reason to expect that physical and biological characteristics of a given oyster bed this year (or month or day) are representative of previous or future years. The ecology of oyster beds, as with other habitat types, changes naturally over time, even in the absence of any human perturbation. Such dynamics often occur over decades, centuries or millennia—time frames that are well beyond the duration of the ‘ecological snapshots’ that are the source of conventional data for assessing the effects of unplanned environmental disturbances [14]. Baseline ecological attributes of an ecosystem, such as an oyster bed, should minimally reflect knowledge of the system on a decadal to centennial scale in order to integrate natural variability [15].

**Figure 1.**
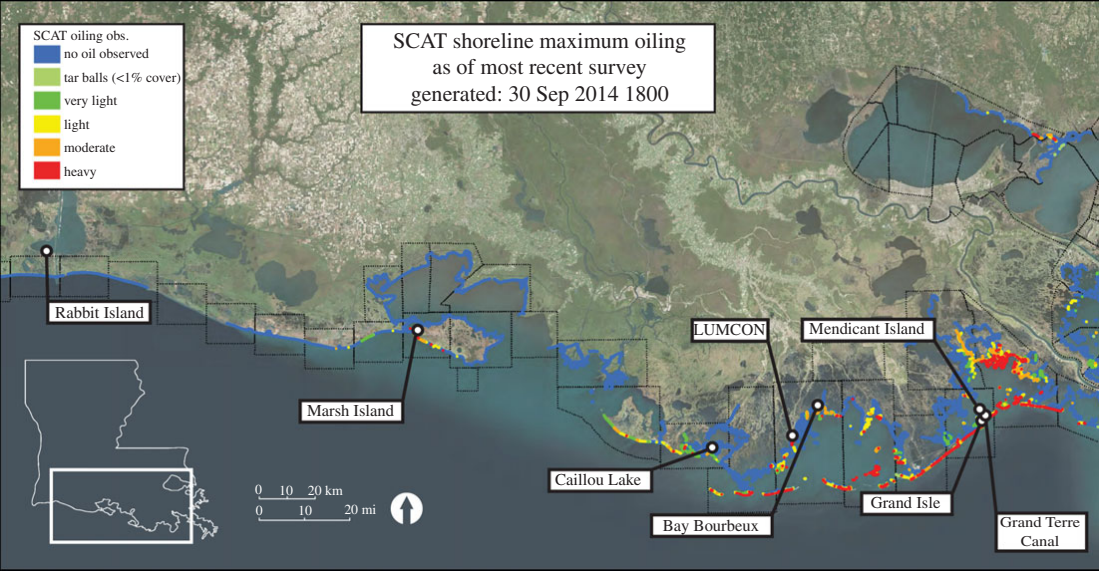
Map showing eight sampling localities along the Louisiana coast. Colour coded shoreline indicates the maximum level of oiling observed by Shoreline Cleanup Assessment Technique (SCAT) teams (Modified from: https://gomex.erma.noaa.gov/layerfiles/19872/files/Reduced_MC252_MaxOilingSituation_Louisiana_30Sep2014.pdf ; accessed 25 August 2016).

Here, we use a novel geohistorical approach to obtain a pre-spill, site-specific, body-size baseline for *C. virginica* from assemblages of dead shells collected from intertidal oyster beds along a 350 km stretch of Louisiana's coastline (figure 1). We compared this baseline with body-size data from live-collected oysters that lived through the DWH oil spill and its aftermath to examine its effects on this iconic estuarine species. We show that average body size of large oysters was not affected by the DWH oil spill.

## 2. Death assemblages

**Figure 2.**
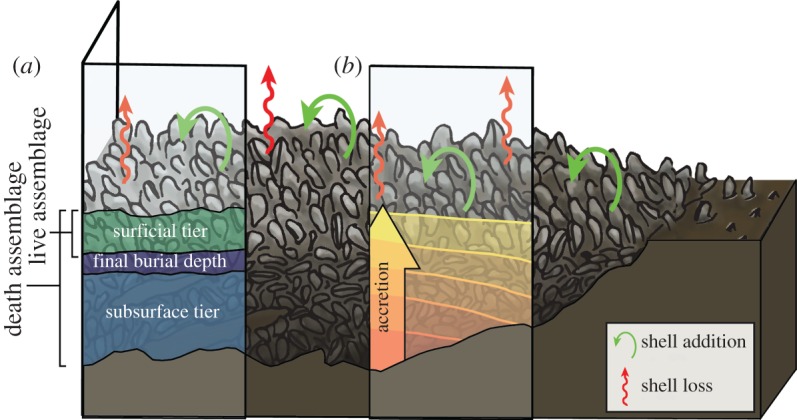
Diagram depicting a hypothetical cross-section of an oyster bed. Red and green arrows represent the processes that continuously remove shell from the bed (e.g. dissolution, breakage) and add it (e.g. new oyster growth). Oyster shells are buried over time by sedimentation and subsidence, producing (*a*) bed structure, including living and dead oysters at the surface (i.e. surficial tier) and dead shells of increasing age with depth in the subsurface tier. As long as shell addition outpaces shell loss, (*b*) accretion (or bed growth) occurs. See text for more details.

Often overlooked sources of long-term baseline data that may be particularly helpful for assessing the DWH oil spill's impacts on oyster populations are death assemblages (DA)—the whole and broken shells that accumulate over time in the uppermost centimetres of the sediment surface on oyster beds (i.e. ‘subsurface tier’ in figure 2; [16]). Most shells in a DA typically are decades to centuries old [17]; thus, oyster DAs capture information on sufficient timescales to average out much of the natural, short-term variation on the oyster bed for a range of ecological attributes [18]. Furthermore, most dead remains never are buried deep enough or are destroyed before being incorporated into the DA, which means that the dead shells buried in oyster beds represent conditions of the bed in the past, relative to the live assemblage (LA; [17]). Quantitative comparisons of ecological attributes such as relative abundance of species in DAs and LAs have been shown to be sensitive to relatively rapid, short-term anthropogenic disturbances [17], because the time-averaged and time-lagged nature of DAs ensures that changes to the LA will take time to be reflected in the composition of the DA. Given these characteristics, oyster DA samples collected before or soon after the oil spill should reflect average conditions on the oyster beds for at least several decades into the past.

## 3. Why oysters?

We focused on oyster beds because, in addition to producing abundant DAs in Louisiana coastal sediments, *C. virginica* is the principal benthic suspension feeder in the northern Gulf of Mexico region [7,19] and provides a wide diversity of ecosystem goods and services, including water filtration, nitrogen removal, provision of habitat for juvenile fish (including many commercially valuable species), stabilization of salt marsh habitats and shorelines, and food production [20,21]. Oysters also have previously been used as ‘ecological indicators’ to assess biological and environmental impacts [22,23] because their epifaunal, sessile life mode makes them both sensitive to contaminants in their environment—including oil [7,24]—and relatively easy to monitor. Given that decreased growth is a common sublethal response in oysters exposed to oil-related contaminants (e.g. [25,26]), we predicted that average body size^1^ of the dead oyster shells (i.e. pre-spill data from DAs) would be larger than the average body size of the live-collected oysters (i.e. post-spill data from LAs) at locations where oil was observed. By contrast, in the absence of oiling (i.e. at control sites), the average body size of live and dead oysters should be similar.

## 4. Material and methods

### 4.1. Sampling and data collection

We sampled dead and live oysters—under Louisiana Department of Wildlife and Fisheries scientific collecting permit # 1915—from eight intertidal sites across Louisiana's coastline that had not previously been subjected to restoration efforts, shell plantings or recent commercial harvesting (figure 1; electronic supplementary material, S1). In addition, to our knowledge, none of the sites we sampled were subjected to clean-up activities related to the DWH oil spill. In 2011, 2012 and/or 2013, each of the localities was sampled within a two-month window (January to February). Our sampling strategy was planned to coincide with the non-spawning period for oysters: 1 year and older oysters typically spawn in the northern Gulf of Mexico from about early spring through early autumn [30].

All locations were representative of oyster beds found in intertidal (less than 1 m tidal amplitude) salt marsh habitats of coastal Louisiana and were subjected to varying levels of direct and indirect impact from the DWH oil spill, as determined by Shoreline Cleanup Assessment Technique (SCAT) surveys of oil-impacted areas (figure 1), but were outside the areas of large-scale impacts of the freshwater diversions from the Mississippi River in Barataria Bay and Black Bay/Breton Sound [13]. The studied salt marshes covered a wide range of expected variability in estuarine habitat attributes—size, surrounding anthropogenic activity and general physical–chemical conditions (e.g. salinity, turbidity). All sites also had similar associated benthic invertebrate species (e.g. presence/absence of mussels, *Ischadium recurvum*, and mud crabs, *Eurypanopeus* spp.).

In each year of sampling, between four and 10 replicate samples were taken at each location. Each sample of live and dead oysters was collected by hand from 30 × 30 cm plots down to approximately 30 cm depth. A steel box with these dimensions was inserted during excavation to maintain the walls of the holes. Dead oysters were only collected in the first year of sampling at each site, because the composition of the DA does not change appreciably from year to year [17]. A 30 cm sampling depth typifies that of many other live–dead datasets derived from marine and estuarine sediments [17]. We followed Powell *et al*. [31] in defining two tiers—surficial and subsurface—in the oyster beds we sampled (figure 2). The surficial tier included living oysters and associated fauna (i.e. the LA) and surficial dead shells (i.e. cultch), and is the layer at which most shell addition and loss takes place (e.g. through dissolution, bioerosion or breakage by predators; [32–36]; figure 2). Half-lives of newly added shells in the surficial tier are usually less than 10 years [35,37]. By contrast, the subsurface tier contains shells that are buried deeply enough that exhumation is unlikely (i.e. they are below the final burial depth; figure 2), and is stable over very long timescales (half-lives for shells in the subsurface tier are at least 50–100 times longer than for shells in the surficial tier; [31]).

A 2.5 mm mesh sieve was used to process each sample. All right valves of live oysters in the surficial tier and dead oysters from the subsurface tier in each sample were sorted and counted, and shell heights (the distance between the shell umbo and growth margin) were measured to the nearest 0.1 mm with digital calipers. Dead shells from the surficial tier samples were excluded. We also limited our live–dead comparisons to large oysters (greater than or equal to 65 mm in shell height) for several reasons. First, because oysters in Louisiana reach 50−60 mm in their first year of growth [38], the large oysters collected in our first year of sampling (2011) must have lived through the DWH oil spill. Second, limiting our analysis to large individuals helped to avoid ambiguous results (see [39] for a similar standardization). For instance, a decrease in mean size in a population could be caused by selective losses of large individuals following an environmental impact (e.g. DWH oil spill) or enhanced recruitment if all individuals are included. Third, large oysters are more durable than smaller oysters, so the larger portion of the size-frequency distribution of a DA best reflects underlying population dynamics of the living population [40].

### 4.2. Data analysis

We used a before-after-control-impact (BACI) analysis [41,42] to test for the effects of the DWH oil spill on oyster body size, which, as an individual-based metric, is one of the best-performing variables for this kind of analysis [43]. Our BACI analysis compared the difference in average oyster body size (greater than or equal to 65 mm in shell height) before and after the oil spill at sites where no oil was observed with the differences at impacted sites. In this analysis, the absence of significant differences between post-spill impact and control site means relative to the pre-spill difference in means would signal recovery (or no impact). Many variants of BACI models exist and are commonly used to assess environmental impacts of both planned developments and unplanned environmental accidents [41,43,44], including other oil spill events in Louisiana [45]. These types of analyses have many advantages, including their ability to handle uneven sampling designs and to account for natural environmental variability among sites [41].

Application of these methods to unplanned environmental accidents is often problematic because data on the indicator of interest from before the accident are not available [41,46]. However, by using the oyster DAs as a pre-spill record of population body size, we were able to avoid this issue. Localities whose sample sites were all located where no visible oil was documented were considered controls, whereas localities having at least one sample site within 200 m of where moderate or heavy oil was observed were considered impacted localities. Based on these criteria, Rabbit Island, Marsh Island, Caillou Lake and Louisiana Universities Marine Consortium (LUMCON) localities were controls and Bay Bourbeux, Grand Isle, Mendicant Island and Grand Terre Canal were impacted localities (figure 1). The BACI analysis was carried out using a mixed model with two fixed factors (treatment and time) and two random factors (locality and year). We included terms for the main effects of treatment (i.e. control or impact) and time (i.e. before or after the DWH oil spill), the interaction between treatment and time, and the random effects of locality and year. We calculated a BACI contrast estimate using the least-squares means for the interaction effect. All statistical analyses were carried out using the statistical package JMP 11.0.0 (SAS Institute Inc.).

## 5. Results

**Figure 3.**
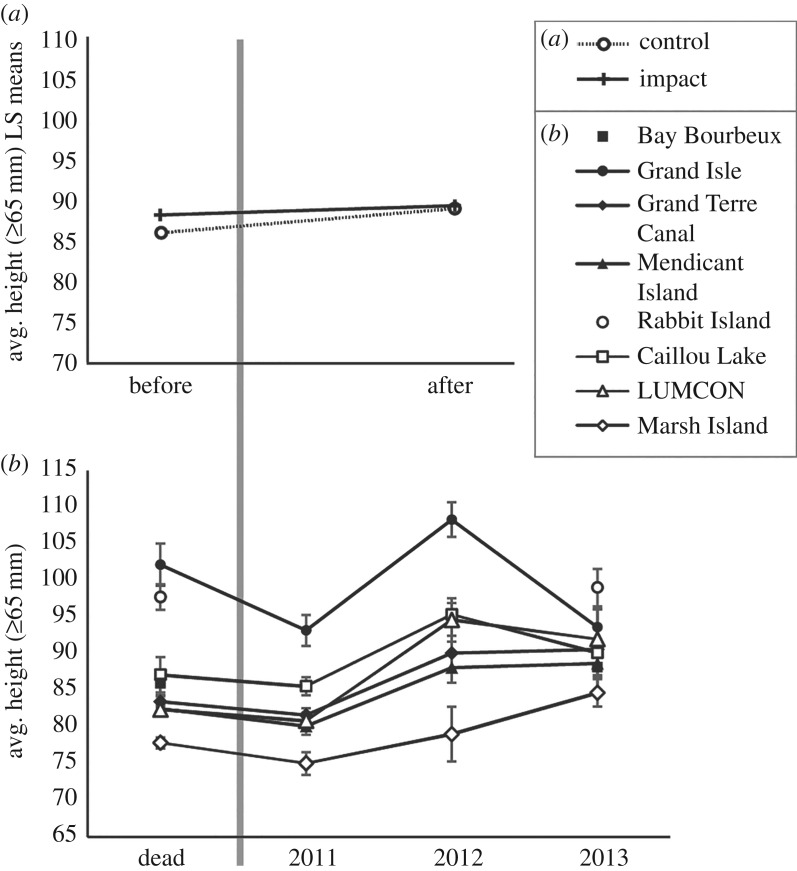
(*a*) Plot showing the least-squares means of the interaction effect from the linear mixed model comparing treatment, time, and treatment × time, and including locality and year as random effects (see text for details). This plot shows a visual representation of the BACI contrast (i.e. the difference in the differences of average body sizes between control and impact localities before and after the spill). The lines for impact and control treatments are nearly parallel, indicating a lack of treatment × time interaction. (*b*) Plot showing trends in average heights of oyster right valves (greater than or equal to 65 mm) from eight localities in Louisiana that either received moderate/high levels of maximum oiling (black shapes; total *n* = 1717) or had no oil observed in the vicinity (white shapes; total *n* = 1919). The vertical grey line indicates data from oysters that lived prior to the DWH spill (before/dead) and those that lived through or recruited following the spill (After/2011–2013). Error bars represent the standard error of the mean.

Results of the linear mixed model—which included more than 3600 live and dead oysters that were collected between 2011 and 2013^2^—showed no significant relationships between treatment (control sites or impacted sites), time (before or after the DWH oil spill) and average body size of oyster right valves greater than or equal to 65 mm in shell height (table 1). The BACI contrast results, conducted on the treatment × time interaction least-squares means, further suggested that there was no significant difference in average size of oysters greater than or equal to 65 mm in shell height between control and impact locations (BACI contrast estimate = 1.9 ± 2.85 s.e., *p* = 0.515; figure 3*a*). Overall, average oyster heights followed similar trends through time at both control and impact locations (figure 3*b*).

**Table 1.** Fixed effects results of a linear mixed model assessing the impact of treatment, time, and their interaction on the average body size of oysters greater than or equal to 65 mm in shell height.

fixed effect	*F*	*p*-value
treatment^a^	*F*_1, 6_._06_ = 0.06	0.812
time^b^	*F*_1, 1_._95_ = 0.13	0.757
treatment × time	*F*_1, 16_._09_ = 0.44	0.515

^a^Control or impact.

^b^Before or after the spill.

## 6. Discussion

Our results suggest that average body size of large oysters (greater than or equal to 65 mm shell height) in intertidal salt marsh habitats of coastal Louisiana remained relatively unchanged after the DWH oil spill, with no differences detected between populations from control and impact locations. In fact, the body-size difference between control and impact locations before and after the oil spill falls well within the variability in average body size of oysters between sites, even within the same treatment category, suggesting that this difference is not biologically significant. For instance, the difference in average body size of oysters between the impacted localities Grand Isle and Mendicant Island in 2011 was 13 mm (electronic supplementary material, S1), several times larger than the difference indicated by the BACI contrast estimate (1.9 mm; figure 3).

Because we conducted the BACI analysis on average body size it was not sensitive to temporal changes in some other potentially important variables [41], such as the shape of the frequency distribution of oyster body sizes before and after the DWH oil spill. However, the distribution of oyster sizes was not different between control and impact treatments for all DA and LA samples (electronic supplementary material, S2). Furthermore, the results did not change when we ran the BACI analysis on median instead of mean oyster body size (electronic supplementary material, S2), suggesting that our focus on means did not mask effects of the DWH spill on the distribution of body sizes.

Furthermore, it is unlikely that our results are biased by among-site variation in environmental factors such as temperature and salinity, which can affect oyster growth and survival both directly (e.g. higher temperature and salinity both tend to increase the growth of oysters; [47]) and indirectly (e.g. pressures from predators and pathogens tend to increase as salinity and temperature increase; [47]). These environmental variables would have had to covary with oiling levels in order to confound the effects of the oil spill on intertidal oyster populations, but there was no systematic variation in either temperature or salinity between our control and impact localities over time, based on data from the Coastwide Reference Monitoring System stations nearest our sampling locations (electronic supplementary material, S1). Another prominent driver of oyster mortality and decreased growth is the pathogen *Perkinsus marinus* (‘Dermo’ disease; [48]). As with the salinity and temperature data, however, Dermo prevalence data from oyster populations near our localities (electronic supplementary material, S1; [49]) did not show patterns with oiling levels that could have masked the DWH oil spill's effects on the studied oyster populations.

Altogether, these results suggest that the DWH oil spill did not affect the body sizes of intertidal oysters in Louisiana. However, our results should not be taken as an indication that oysters were not exposed to (or consumed) oil-derived materials. Rather, they imply that oyster populations either recovered rapidly following the immediate impact of the spill, or simply that the DWH oil spill's impact on intertidal oyster beds in Louisiana was not severe enough to influence oyster population dynamics in the short term.

### 6.1. Other studies of oyster response to the Deepwater Horizon oil spill

Our findings of little impact of the DWH oil spill on intertidal oysters in Louisiana are consistent with other studies of oyster populations [9,50]. For instance, Carmichael *et al*. [50] conducted experimental transplants of hatchery-reared oysters along the Mississippi–Alabama coastline, and found that oyster growth was not significantly affected by potential oil exposure. Also, in Barataria Bay and Breton Sound in Louisiana, La Peyre *et al*. [9] found that polycyclic aromatic hydrocarbon (PAH) concentrations were not elevated to levels that would be expected to harm oysters. Xia *et al*. [51] presented similar findings from Mississippi, showing that elevated PAH concentrations in oyster tissues following the spill were low overall, and declined relatively rapidly, averaging 34 ng g^−1^ (dry weight) in July and August 2010, and falling to an average of 15 ng g^−1^ by September 2010 (although these levels may be high enough to cause sublethal effects in marine invertebrates in some cases; see [52]). Likewise, in a study of oysters from Lake Borgne and Mississippi Sound, Louisiana, Soniat *et al*. [22] found no evidence of PAH contamination in tissue samples analysed six months after the DWH oil spill, and a Natural Resource Damage Assessment report also found that PAH concentrations in oyster tissues following the DWH oil spill were not elevated above baseline values measured before the spill [53].

These findings and ours do not imply, however, that the DWH oil spill had no effect on oyster populations. For instance, oyster densities were lower in 2010–2013 in some regions affected by the DWH oil spill relative to pre-spill baseline information from 2006 to 2009, whereas no difference relative to 2006–2009 baseline data were observed in regions unaffected by the DWH oil spill [11,54]. Furthermore, a variety of emergency response activities resulted in locally severe oyster habitat damage and mortality [10–13]. In particular, per cent cover of oyster shell in near shore habitats (within 50 m of the marsh edge) was reduced, both as a function of the severity of oiling and whether the area was treated to remove oil [10]. Also, the freshwater diversions from the Mississippi River, which were conducted as part of the emergency response, reduced average salinities to less than 5 ppt across vast portions of northern Barataria Bay and Black Bay/Breton Sound in Louisiana for more than 30 consecutive days, killing at least 1.1 billion adult-equivalent subtidal oysters [13]. Oyster populations in the areas affected by the diversions are not expected to recover before 2017, due to the combination of direct oyster mortality and the resulting depressed recruitment, and habitats where sedimentation or oil clean-up activities made substrates unsuitable for spat settlement may not recover at all without restoration action [10,12].

### 6.2. Potential factors explaining why oyster body size did not change following the Deepwater Horizon oil spill

Despite locally severe impacts of the DWH oil spill on oyster populations, overall we are faced with the counterintuitive result that the largest marine oil spill in US history apparently did not have widespread effects on many intertidal oyster populations, even those in close proximity to locations that were heavily oiled. Although identifying the cause of this result is beyond the scope of our study, one or more factors may help explain it.

First, impacts from the DWH oil spill may have been less than expected because the oil was released at 1.6 km depth and 80 km from the shore. This great distance gave the surface oil time to at least partially degrade before reaching the coast [1,55], limiting its toxicity when (if) ingested by oysters. Second, although some studies have suggested that oil contamination may decrease growth in oysters [25,26,56,57], others have found little or no effect of hydrocarbons on growth [58], suggesting that impacts to Gulf Coast oysters from the DWH oil spill may have been limited. In particular, extensive experimental and field studies designed to assess the impact of the oil and gas industry on Gulf Coast oysters, conducted between the late 1940s and early 1960s—known colloquially as Projects 9 and 23—repeatedly found that the effects of light to moderate oil contamination and bleedwater disposal on oyster recruitment, growth and mortality were negligible [59,60].

These experimental observations raise the interesting possibility that there may be inherent differences in the physiologies of oysters living along the coast of Louisiana—due to their proximity to naturally occurring oil inputs from hydrocarbon seeps in the northern Gulf of Mexico [61]—that make them more tolerant to oil contamination than conspecifics from regions where natural hydrocarbon ‘pollution’ is uncommon. Indeed, increased tolerance to oil has been observed in other sessile, epifaunal marine bivalve species inhabiting waters with naturally persistent exposure to oil [62,63]. For instance, Kanter *et al*. [63] found that the mussel *Mytilus californianus* living near natural oil seeps experienced less mortality in several treatments involving various concentrations of oil than conspecifics collected from locations without natural exposure to oil. They pointed out that their results might help explain why mortality among rocky intertidal fauna following an oil spill in 1969 in the Santa Barbara Channel (near natural oil seeps) was lower than expected [63]. Some evidence from studies of microbial communities associated with oysters suggests populations of oysters may have similar tolerances to oil exposure. Although relatively little is known about their function, several symbiotic bacterial species capable of degrading oil have been isolated from tissue and mantle fluids of Gulf Coast oysters [64,65]. These bacteria could play a role in enhancing an oyster host's resistance to the adverse effects of toxins from consumed oil-derived materials [66]. If *C. virginica* benefits from their symbiotic microbiota in this way, then populations living in Louisiana may have been pre-adapted to cope with short-term stress related to pulses of hydrocarbon contamination such as occurred following the DWH oil spill.

Third, population-level responses to disturbances can yield counterintuitive results when compared with the results of individual-level studies. For instance, Fodrie *et al*. [67] pointed out that although studies of hydrocarbon toxicity in fish have often demonstrated deleterious effects, most population-level studies have failed to identify negative impacts related to oil contamination. They suggested several possible causes for this pattern that may also be applicable to oyster populations. (i) Fishery closures related to the DWH oil spill could have benefitted populations of many commercially valuable species of finfish and shellfish, possibly including oysters, by decreasing mortality and allowing increased recruitment. (ii) If effects elsewhere in the food web decreased abundances of various predators of oysters and/or oyster larvae, then oyster populations may have largely kept up with the negative effects of oiling. For instance, barnacles can be important predators of oyster larvae and are thought to be vulnerable to hydrocarbon pollution [60], although there is also evidence to the contrary [52,68]. (iii) Populations are often capable of responding to declining abundance with compensatory production, due, for instance, to greater survival of larvae or juveniles, that can largely make up for high mortality, and there is evidence that this may be the case for oysters in Louisiana [38]. And, (iv) sublethal and chronic effects of oil contamination may take several years to become detectable [67]. For instance, the collapse of the Pacific herring population in Prince William Sound was not apparent until over 4 years after the *Exxon Valdez* oil spill [69]. Sublethal population effects of oil contamination can also persist in marshes for long periods of time [70,71]; for instance, residual oil contamination in marsh sediments from the *Florida* oil spill in Buzzards Bay, Massachusetts, in 1969 was still reducing growth rates and overall sizes of local ribbed mussels, *Geukensia demissa*, nearly four decades later [71]. Indeed, sublethal effects on critical physiological processes in marine invertebrates, including reproduction, growth, respiration and many others can result from oil concentrations as low as 1−10 ppb, depending on the chemical characteristics of the oil [52].

Thus, it is possible that it will still be years before the full extent of the DWH oil spill's effects on oyster beds in Louisiana are fully realized. However, the responses discussed above are not all mutually exclusive, and it is possible that properties of Louisiana oyster microbiomes, fisheries closures, differential mortality of oyster predators, and/or capacity for compensatory production in Louisiana oyster populations could all have contributed to the apparent lack of impact of the DWH spill observed in our study. Further research will be required to differentiate between these alternatives.

### 6.3. The advantage of geohistorical records for environmental assessment

Finally, our study took a novel step forward methodologically. To the best of our knowledge, this study is the first to conduct a BACI analysis using baseline data from geohistorical records. Our results highlight the advantage of using geohistorical records to establish baselines, even multiple years after an environmental disturbance. The unique time-averaged and time-lagged properties of DAs cause them to record baseline characteristics of a location over long timescales (i.e. decades to centuries). Thus, the use of data from DAs and other geohistorical records shows promise for addressing the impacts of unplanned environmental disasters where local baseline data are scarce.

## Supplementary Material

Electronic supplementary material 1: Locality and site descriptions

## Supplementary Material

Electronic supplementary material 2: Analysis of oyster body size distributions

## Footnotes

1 We measured body size because it is one of the most important functional traits of an organism, which influences the type and strength of ecological interactions [27] and life-history traits [28]. Furthermore, many ecological processes also are strongly size-dependent; that is, size-based metrics integrate a variety of information on the state and functioning of ecosystems. Body size also can be measured from both live and dead oysters, is comparable across sites and is expected to vary along disturbance gradients according to ecological and energetic constraints [29].

2 See electronic supplementary material, S1 for a breakdown of oyster abundance data by sampling site and year.

## Data accessibility

Body-size data are available from the Dryad Digital Repository at http://dx.doi.org/10.5061/dryad.bc80t [72].

## References

[1] Deepwater Horizon Natural Resource Damage Assessment Trustees. 2016 Deepwater Horizon oil spill: final programmatic damage assessment and restoration plan and final programmatic environmental impact statement. Retrieved from http://www.gulfspillrestoration.noaa.gov/restoration-planning/gulf-plan . (accessed 28 February 2016).

[2] Michel J et al. 2013 Extent and degree of shoreline oiling: Deepwater Horizon oil spill, Gulf of Mexico, USA. PLoS ONE 8, e65087 (doi:10.1371/journal.pone.0065087) 23776444 10.1371/journal.pone.0065087 PMC3680451

[3] Zengel S, Bernik BM, Rutherford N, Nixon Z, Michel J 2015 Heavily oiled salt marsh following the Deepwater Horizon oil spill, ecological comparisons of shoreline cleanup treatments and recovery. PLoS ONE 10, e0132324 (doi:10.1371/journal.pone.0132324) 26200349 10.1371/journal.pone.0132324 PMC4511762

[4] Powers SP, Scyphers SB 2016 *Estimating injury to nearshore fauna resulting from the Deepwater Horizon oil spill*. DWH Oyster NRDA Technical Working Group Report, 57 p.

[5] Silliman BR, van de Koppel J, McCoy MW, Diller J, Kasozi GN, Earl K, Adams PN, Zimmerman AR 2012 Degradation and resilience in Louisiana salt marshes after the BP-Deepwater Horizon oil spill. Proc. Natl Acad. Sci. USA 109, 11 234–11 239. (doi:10.1073/pnas.1204922109) 10.1073/pnas.1204922109 PMC3396483 22733752

[6] Rabalais N 2014 Assessing early looks at biological responses to the Macondo event. BioScience 64, 757–759. (doi:10.1093/biosci/biu132)

[7] Mendelssohn IA et al. 2012 Oil Impacts on coastal wetlands: implications for the Mississippi River delta ecosystem after the *Deepwater Horizon* oil spill. BioScience 62, 562–574. (doi:10.1525/bio.2012.62.6.7)

[8] Sumaila UR et al. 2012 Impact of the *Deepwater Horizon* well blowout on the economics of US Gulf fisheries. Can. J. Fish. Aquat. Sci. 69, 499–510. (doi:10.1139/f2011-171)

[9] La Peyre JF, Casas SM, Miles S 2014 Oyster responses to the Deepwater Horizon oil spill across coastal Louisiana: examining oyster health and hydrocarbon bioaccumulation. In Impacts of oil spill disasters on marine habitats and fisheries in North America (eds Alford JB, Peterson MS, Green CC), pp. 269–294. Boca Raton, FL: CRC Press.

[10] Powers SP, Rouhani S, Baker MC, Roman H, Murray J, Grabowski JH, Scyphers SB, Willis JM, Hester MW 2015 *Loss of oysters as a result of the Deepwater Horizon oil spill degrades nearshore ecosystems and disrupts facilitation between oysters and marshes*. DWH Oyster NRDA Technical Working Group Report, 11 p.

[11] Grabowski JH, Powers SP, Roman H, Rouhani S 2015 *Impacts of the 2010 Deepwater Horizon oil spill and associated response activities on subtidal oyster populations in the northern Gulf of Mexico*. DWH Oyster NRDA Technical Working Group Report, 12 p.

[12] Grabowski JH, Marrison HM, Murray J, Roman H, Rouhani S, Powers SP 2015 *Technical memorandum: oyster recruitment failure in the northern Gulf of Mexico as a consequence of the 2010 Deepwater Horizon oil spill*. DWH Oyster NRDA Technical Working Group Report, 18 p.

[13] Powers SP, Grabowski JH, Roman H, Geggel A, Rouhani S, Oehrig J, Baker M 2015 *Consequences of large scale hydrographic alteration during the Deepwater Horizon oil spill on subtidal oyster populations*. DWH Oyster NRDA Technical Working Group Report, 15 p.

[14] McClenachan L, Ferretti F, Baum JK 2012 From archives to conservation: why historical data are needed to set baselines for marine animals and ecosystems. Conserv. Lett. 5, 349–359. (doi:10.1111/j.1755-263X.2012.00253.x)

[15] Swetnam TW, Allen CD, Betancourt JL 1999 Applied historical ecology: using the past to manage for the future. Ecol. Appl. 9, 1189–1206. (doi:10.1890/1051-0761(1999)009[1189:AHEUTP]2.0.CO;2)

[16] Dietl GP, Flessa KW 2011 Conservation paleobiology: putting the dead to work. Trends Ecol. Evol. 26, 30–37. (doi:10.1016/j.tree.2010.09.010) 21035892 10.1016/j.tree.2010.09.010

[17] Kidwell SM 2009 Evaluating human modification of shallow marine ecosystems: mismatch in composition of molluscan living and time-averaged death assemblages. In Conservation paleobiology: using the past to manage for the future (eds Dietl GP, Flessa KW), pp. 113–139. New Haven, CT: Yale University Printing and Publishing Services.

[18] Kowalewski M, Goodfriend GA, Flessa KW 1998 High-resolution estimates of temporal mixing within shell beds: the evils and virtues of time-averaging. Paleobiology 24, 287–304.

[19] Kilgen RH, Dugas RJ 1989 *The ecology of oyster reefs of the northern Gulf of Mexico: an open file report*. National Wetlands Research Center Open File Report 89-03. U.S. Fish and Wildlife Service, 113 p.

[20] Grabowski JH, Peterson CH 2007 Restoring oyster reefs to recover ecosystem services. In Ecosystem engineers: concepts, theory and applications (eds Cuddington K, Byers JE, Wilson WG, Hastings A), pp. 281–298. Amsterdam, The Netherlands: Elsevier.

[21] Coen LD, Brumbaugh RD, Bushek D, Grizzle R, Luckenbach MW, Posey MH, Powers SP, Tolley SG 2007 Ecosystem services related to oyster restoration. Mar. Ecol. Prog. Ser. 341, 303–307. (doi:10.3354/meps341303)

[22] Soniat TM, King SM, Tarr MA, Thorne MA 2011 Chemical and physiological measures on oysters (*Crassostrea virginica*) from oil-exposed sites in Louisiana. J. Shellfish Res. 30, 713–717. (doi:10.2983/035.030.0311)

[23] Volety AK, Savarese M, Tolley SG, Arnold WS, Sime P, Goodman P, Chamberlain RH, Doering PH 2009 Eastern oysters (*Crassostrea virginica*) as an indicator for restoration of Everglades ecosystems. Ecol. Indic. 9, S120–S136. (doi:10.1016/j.ecolind.2008.06.005)

[24] Law RJ, Kelly C, Baker K, Jones J, McIntosh AD, Moffat CF 2002 Toxic equivalency factors for PAH and their applicability in shellfish pollution monitoring studies. J. Environ. Monit. JEM 4, 383–388. (doi:10.1039/b200633m) 12094932 10.1039/b200633m

[25] Mahoney BMS, Noyes GS 1982 Effects of petroleum on feeding and mortality of the American oyster. Arch. Environ. Contam. Toxicol. 11, 527–531. (doi:10.1007/BF01056358)

[26] Capuzzo JM 1996 The bioaccumulation and biological effects of lipophilic organic contaminants. In The eastern oyster, Crassostrea virginica (eds Kennedy VS, Newell RIE, Eble AF), pp. 539–557. College Park, MD: Maryland Sea Grant College.

[27] Jennings S, Mackinson S 2003 Abundance–body mass relationships in size-structured food webs. Ecol. Lett. 6, 971–974. (doi:10.1046/j.1461-0248.2003.00529.x)

[28] Shin Y, Rochet M, Jennings S, Field J, Gislason H 2005 Using size-based indicators to evaluate the ecosystem effects of fishing. ICES J. Mar. Sci. 62, 384–396. (doi:10.1016/j.icesjms.2005.01.004)

[29] Woodward G, Ebenman B, Emmerson M, Montoya JM, Olesen JM, Valido A, Warren PH 2005 Body size in ecological networks. Trends Ecol. Evol. 20, 402–409. (doi:10.1016/j.tree.2005.04.005) 16701403 10.1016/j.tree.2005.04.005

[30] Hayes PF, Menzel RW 1981 The reproductive cycle of early setting *Crassostrea virginica* (Gmelin) in the northern Gulf of Mexico, and its implications for population recruitment. Biol. Bull. 160, 80–88. (doi:10.2307/1540902)

[31] Powell EN, Klinck JM, Ashton-Alcox K, Hofmann EE, Morson J 2012 The rise and fall of *Crassostrea virginica* oyster reefs: the role of disease and fishing in their demise and a vignette on their management. J. Mar. Res. 70, 505–558. (doi:10.1357/002224012802851878)

[32] Carriker MR, Palmer RE, Prezant RS 1980 Functional ultramorphology of the dissoconch valves of the oyster *Crassostrea virginica*. Proc. Natl Shellfish. Assoc. 70, 139–183.

[33] Christmas JF, McGinty MR, Randle DA, Smith GF, Jordan SJ 1997 Oyster shell disarticulation in three Chesapeake Bay tributaries. J. Shellfish Res. 16, 115–123.

[34] Carver CE, Thériault I, Mallet AL 2010 Infection of cultured eastern oysters *Crassostrea virginica* by the boring sponge *Cliona celata*, with emphasis on sponge life history and mitigation strategies. J. Shellfish Res. 29, 905–915. (doi:10.2983/035.029.0423)

[35] Powell EN, Kraeuter JN, Ashton-Alcox KA 2006 How long does oyster shell last on an oyster reef? Estuar. Coast. Shelf Sci. 69, 531–542. (doi:10.1016/j.ecss.2006.05.014)

[36] Elner RW, Lavoie RE 1983 Predation on American oysters (*Crassostrea virginica* [Gmelin]) by American lobsters (*Homarus americanus* Milne-Edwards), rock crabs (*Cancer irroratus* Say), and mud crabs (*Neopanope sayi* [Smith]). J. Shellfish Res. 3, 129–134.

[37] Waldbusser GG, Steenson RA, Green MA 2011 Oyster shell dissolution rates in estuarine waters: effects of pH and shell legacy. J. Shellfish Res. 30, 659–669. (doi:10.2983/035.030.0308)

[38] Casas SM, La Peyre J, La Peyre MK 2015 Restoration of oyster reefs in an estuarine lake: population dynamics and shell accretion. Mar. Ecol. Prog. Ser. 524, 171–184. (doi:10.3354/meps11198)

[39] Marchini A, Caronni S, Occhipinti-Ambrogi A 2008 Size variations of the amphipod crustacean *Melita palmata* in two Adriatic lagoons: Goro and Lesina. Transitional Waters Bull. 2, 1–12.

[40] Cummins H, Powell EN, Stanton RJ Jr, Staff G 1986 The size-frequency distribution in palaeoecology: effects of taphonomic processes during formation of molluscan death assemblages in Texas bays. Palaeontology 29, 495–518.

[41] Underwood AJ 1994 On beyond BACI: sampling designs that might reliably detect environmental disturbances. Ecol. Appl. 4, 4–15. (doi:10.2307/1942110)

[42] Stewart-Oaten A, Murdoch WW, Parker KR 1986 Environmental impact assessment: ‘pseudoreplication’ in time? Ecology 67, 929–940. (doi:10.2307/1939815)

[43] Osenberg CW, Schmitt RJ, Holbrook SJ, Abu-Saba KE, Flegal AR 1996 Detection of environmental impacts: natural variability, effect size, and power analysis. In Detecting ecological impacts: concepts and applications in coastal habitats (eds Schmitt RJ, Osenberg CW), pp. 83–108. New York, NY: Academic Press.

[44] Smith EP, Orvos DR, Cairns J Jr 1993 Impact assessment using the before-after-control-impact (BACI) model: concerns and comments. Can. J. Fish. Aquat. Sci. 50, 627–637. (doi:10.1139/f93-072)

[45] Roth A-MF, Baltz DM 2009 Short-term effects of an oil spill on marsh-edge fishes and decapod crustaceans. Est. Coasts 32, 565–572. (doi:10.1007/s12237-009-9135-2)

[46] Martinez-Gomez C, Vethaak AD, Hylland K, Burgeot T, Kohler A, Lyons BP, Thain J, Gubbins MJ, Davies IM 2010 A guide to toxicity assessment and monitoring effects at lower levels of biological organization following marine oil spills in European waters. ICES J. Mar. Sci. J. Conserv. 67, 1105–1118. (doi:10.1093/icesjms/fsq017)

[47] Shumway SE 1996 Natural environmental factors. In The eastern oyster, Crassostrea virginica (eds Kennedy VS, Newell RIE, Eble AF), pp. 467–513. College Park, MD: Maryland Sea Grant College.

[48] Ford SE, Tripp MR 1996 Diseases and defense mechanisms. In The eastern oyster, Crassostrea virginica (eds Kennedy VS, Newell RIE, Eble AF), pp. 581–660. College Park, MD: Maryland Sea Grant College.

[49] Soniat TM, Ray SM 2011 *Perkinsus marinus* (accessed 17 September 2016). See http://oystersentinel.org/?q=perkinsus.

[50] Carmichael RH, Jones AL, Patterson HK, Walton WC, Pérez-Huerta A, Overton EB, Dailey M, Willett KL 2012 Assimilation of oil-derived elements by oysters due to the Deepwater Horizon oil spill. Environ. Sci. Technol. 46, 12 787–12 795. (doi:10.1021/es302369h) 10.1021/es302369h 23131011

[51] Xia K, Hagood G, Childers C, Atkins J, Rogers B, Ware L 2012 Polycyclic aromatic hydrocarbons (PAHs) in Mississippi seafood from areas affected by the Deepwater Horizon oil spill. Environ. Sci. Technol. 46, 5310–5318. (doi:10.1021/es2042433) 22524970 10.1021/es2042433

[52] Suchanek TH 1993 Oil impacts on marine invertebrate populations and communities. Am. Zool. 33, 510–523. (doi:10.1093/icb/33.6.510)

[53] Oehrig J, Rouhani S, Zhang M 2015 *Summary of Deepwater Horizon trustees NRDA nearshore tissue tPAH concentrations*. DWH Oyster NRDA Technical Working Group Report, 12 p.

[54] Roman H 2015 *Technical memorandum: development of oyster nearshore injury quantification*. DWH Oyster NRDA Technical Working Group Report, 7 p.

[55] Liu Z, Liu J, Zhu Q, Wu W 2012 The weathering of oil after the Deepwater Horizon oil spill: insights from the chemical composition of the oil from the sea surface, salt marshes and sediments. Environ. Res. Lett. 7, 35302 (doi:10.1088/1748-9326/7/3/035302)

[56] Rabalais NN, McKee BA, Reed DJ, Means JC 1992 Fate and effects of produced water discharges in coastal Louisiana, Gulf of Mexico, USA. In Produced Water (eds Ray JP, Engelhart FR), pp. 355–369. New York, NY: Plenum Press.

[57] National Research Council (U.S.). 2003 Oil in the sea III: inputs, fates, and effects. Washington, DC: National Academy Press, 265 p. 25057607

[58] Galtsoff PS, Prytherch HF, Smith RO, Koehring V 1935 Effects of crude oil pollution on oysters in Louisiana waters. Bull. Bur. Fish. 48, 143–210.

[59] Mackin JG, Hopkins SH 1961 Studies on oyster mortality in relation to natural environments and to oil fields in Louisiana. Publ. Inst. Mar. Sci. 7, 1–131.

[60] Tunnell JW 2011 *An expert opinion of when the Gulf of Mexico will return to pre-spill harvest status following the BP Deepwater Horizon MC 252 oil spill*. Report for the Gulf Coast Claims Facility. Washington, DC, 52p.

[61] Kvenvolden KA, Cooper CK 2003 Natural seepage of crude oil into the marine environment. Geo-Mar. Lett. 23, 140–146. (doi:10.1007/s00367-003-0135-0)

[62] Straughan D 1977 Effects of natural chronic exposure to petroleum hydrocarbons on size and reproduction in *Mytilus californianus* Conrad. In Physiological responses of marine biota to pollutants (eds Vernberg FJ, Calabrese A, Thurberg FP, Vernberg WB), pp. 289–298. New York, NY: Academic Press.

[63] Kanter R, Straughan D, Jessee WN 1971 Effects of exposure to oil on *Mytilus californianus* from different localities. In *Proceedings of Joint Conference on Prevention and Control of Oil Spills, Washington, DC, 15–17 June*, pp. 485–488. Washington, DC: American Petroleum Institute. (http://dx.doi.org/10.7901/2169-3358-1971-1-485)

[64] Thomas JC, Wafula D, Chauhan A, Green SJ, Gragg R, Jagoe C 2014 A survey of Deepwater Horizon (DWH) oil-degrading bacteria from the eastern oyster biome and its surrounding environment. Aquat. Microbiol. 5, 149 (doi:10.3389/fmicb.2014.00149) 10.3389/fmicb.2014.00149 PMC3988384 24782841

[65] King GM, Judd C, Kuske CR, Smith C 2012 Analysis of stomach and gut microbiomes of the eastern oyster (*Crassostrea virginica*) from coastal Louisiana, USA. PLoS ONE 7, e51475 (doi:10.1371/journal.pone.0051475) 23251548 10.1371/journal.pone.0051475 PMC3520802

[66] Harris JM 1993 The presence, nature, and role of gut microflora in aquatic invertebrates: a synthesis. Microb. Ecol. 25, 195–231. (doi:10.1007/BF00171889) 24189919 10.1007/BF00171889

[67] Fodrie FJ, Able KW, Galvez F, Heck KL, Jensen OP, Lopez-Duarte PC, Martin CW, Turner RE, Whitehead A 2014 Integrating organismal and population responses of estuarine fishes in Macondo spill research. BioScience 64, 778–788. (doi:10.1093/biosci/biu123)

[68] Fry B, Anderson LC 2014 Minimal incorporation of Deepwater Horizon oil by estuarine filter feeders. Mar. Pollut. Bull. 80, 282–287. (doi:10.1016/j.marpolbul.2013.10.018) 24461698 10.1016/j.marpolbul.2013.10.018

[69] Thorne RE, Thomas GL 2008 Herring and the ‘Exxon Valdez’ oil spill: an investigation into historical data conflicts. ICES J. Mar. Sci. J. Cons. 65, 44–50. (doi:10.1093/icesjms/fsm176)

[70] Fukuyama AK, Shigenaka G, Hoff RZ 2000 Effects of residual Exxon Valdez oil on intertidal *Protothaca staminea*: mortality, growth, and bioaccumulation of hydrocarbons in transplanted clams. Mar. Pollut. Bull. 40, 1042–1050. (doi:10.1016/S0025-326X(00)00055-2)

[71] Culbertson JB, Valiela I, Olsen YS, Reddy CM 2008 Effect of field exposure to 38-year-old residual petroleum hydrocarbons on growth, condition index, and filtration rate of the ribbed mussel, *Geukensia demissa*. Environ. Pollut. 154, 312–319. (doi:10.1016/j.envpol.2007.10.008) 18045755 10.1016/j.envpol.2007.10.008

[72] Dietl GP, Durham SR 2016 Data from: geohistorical records indicate no impact of the Deepwater Horizon oil spill on oyster body size. Dryad Digital Repository. (http://dx.doi.org/10.5061/dryad.bc80t )

