# Supplementary material for: Geohistorical records indicate no impact of the Deepwater Horizon oil spill on oyster body size
Source: R Soc Open Sci. 2016 Nov 30;3(11):160763. doi: 10.1098/rsos.160763 (PMC5180161; doi:10.1098/rsos.160763)
Supplement: Electronic supplementary material 1: Locality and site descriptions [file rsos160763supp1.pdf]

Geohistorical records indicate no impact of the Deepwater Horizon oil spill on oyster body size

Gregory P. Dietl, Stephen R. Durham

Electronic supplementary material S1: Locality and site descriptions

This document describes each locality sampled for our study of oyster population body size changes in response to the Deepwater Horizon (DWH) oil spill from 2011 to 2013. The eight localities were spread across approximately 350 km of Louisiana's coastline from Calcasieu Lake to Barataria Bay and included both areas that received oil from the DWH spill and areas where oil did not make landfall. The localities were sampled in winter or early spring each year to ensure that variability in population size structure from season to season did not affect our results. Locality selection was based on the presence of intertidal oysters, the lease-status of the sampling areas (only areas outside fishing leases and known restoration areas were sampled), and the documented maximum oiling observed from Shoreline Cleanup and Assessment Technique (SCAT) surveys. Color-coded SCAT survey results as of 9/30/2014 are shown on the map at the bottom of this page, along with numbered icons corresponding to the positions of our sampling localities along the Louisiana shoreline (Fig. S1.1). All surface samples were collected from a randomly placed 30 x 30 cm quadrat. In the first year of sampling at each locality, the dead shells buried beneath the surface of the oyster beds, called death assemblages, were also sampled to a depth of 30cm to provide pre-spill, time-averaged, population body-size baselines (Fig. S1.2). Sample sites at each locality were recorded with handheld GPS in 2011 and 2013, but sample locations for 2012 are estimated from field notes and photos.

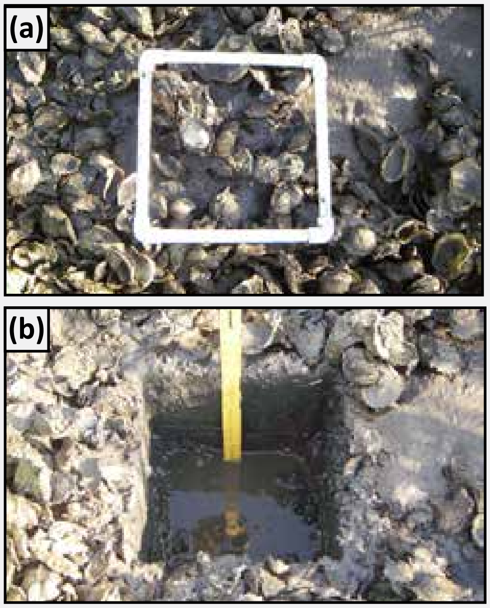

Figure S1.2. Photographs showing an example of a 30 x 30 cm sample plot (a) before and (b) after collection of both the surface layer and the 30 cm death assemblage samples. Photos are of Site 7 from Grand Terre Canal in 2011.

Figure S1.1. Table of contents and map showing maximum oil levels observed by Shoreline Cleanup and Assessment Technique (SCAT) surveys along a section of the Louisiana coastline as of 9/30/2014. Numbered circles indicate localities sampled for the present study. Map is modified from: https://gomex.erma.noaa.gov/layerfiles/19872/files/Reduced_MC252_MaxOilingSituation_Louisiana_30Sep2014.pdf; accessed 8/25/2016.

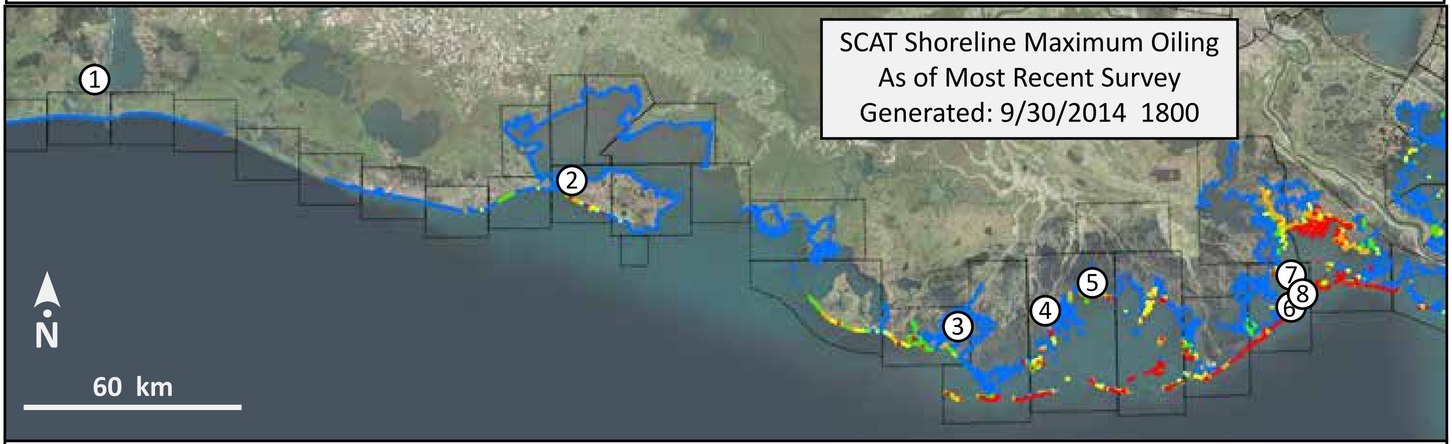

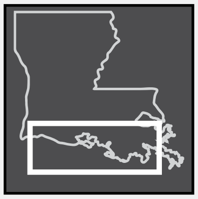

Localities	Page		Page
1. Rabbit Island	2	5. Bay Bourbeux	18
2. Marsh Island	6	6. Grand Isle	22
3. Caillou Lake	10	7. Mendicant Island	26
4. LUMCON	14	8. Grand Terre Canal	30

SCAT Oiling Obs.

- No Oil Observed
- Tar Balls (< 1% cover)
- Very Light
- Light
- Moderate
- Heavy

The western-most sites were located on Rabbit Island, in the southwestern portion of Calcasieu Lake (Figs. S1.3, S1.7). This small (~1 km across), marsh island is intruded by tidal channels and mud flats, where we sampled six fringing intertidal oyster beds. This locality was categorized as a control area because SCAT surveys observed no oil on Rabbit Island (Fig. S1.4). The locality was sampled once, in 2013, and both surface and death assemblage samples were collected (Table S1.1). Oyster right valves ≥65 mm in height were abundant in both the living and death assemblages at this location (Table S1.2), and there was very little difference in the average heights between the two assemblages (Fig. S1.5).

Environmental data from the nearest Coastwide Reference Monitoring System (CRMS) station to the sampling sites (CRMS 0685: 29°53’23.28”N, 93°22’11.11”W; ~4.5 km north of Rabbit Island) showed that between 2006–2013, average water temperature ± se during the summer months, May–August, was 28.3ºC ± 0.72. Average salinity ± se over the same interval was 15.2‰ ± 0.42. Data on the prevalence of *Perkinsus marinus* (dermo disease) infection from the nearest Oyster Sentinel station to our sampling sites (N. E. Rabbit Island: 29°51’25.92”N, 93°21’47.08”W; ~1.75 km northeast of our Rabbit Island sampling sites) indicated that average dermo prevalence ± se near Rabbit Island was 0.63 ± 0.15 between 2006 and 2013 (although no data were available for 2009). All environmental and dermo prevalence data were accessed from www.lacoast.gov/crms and www.oystersentinel.org, respectively (Fig. S1.6).

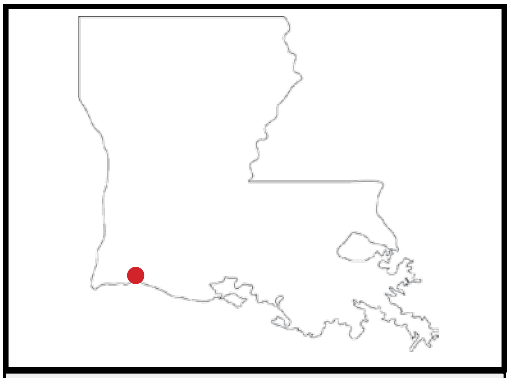

Figure S1.3. Map showing the location (red dot) of Rabbit Island in Louisiana.

Year	DA	LA		
	2013	2011	2012	2013
Samples	6	NA	NA	6

Table S1.1. Table showing the year(s) Rabbit Island was sampled and the number of samples collected. DA = death assemblage; LA = live assemblage.

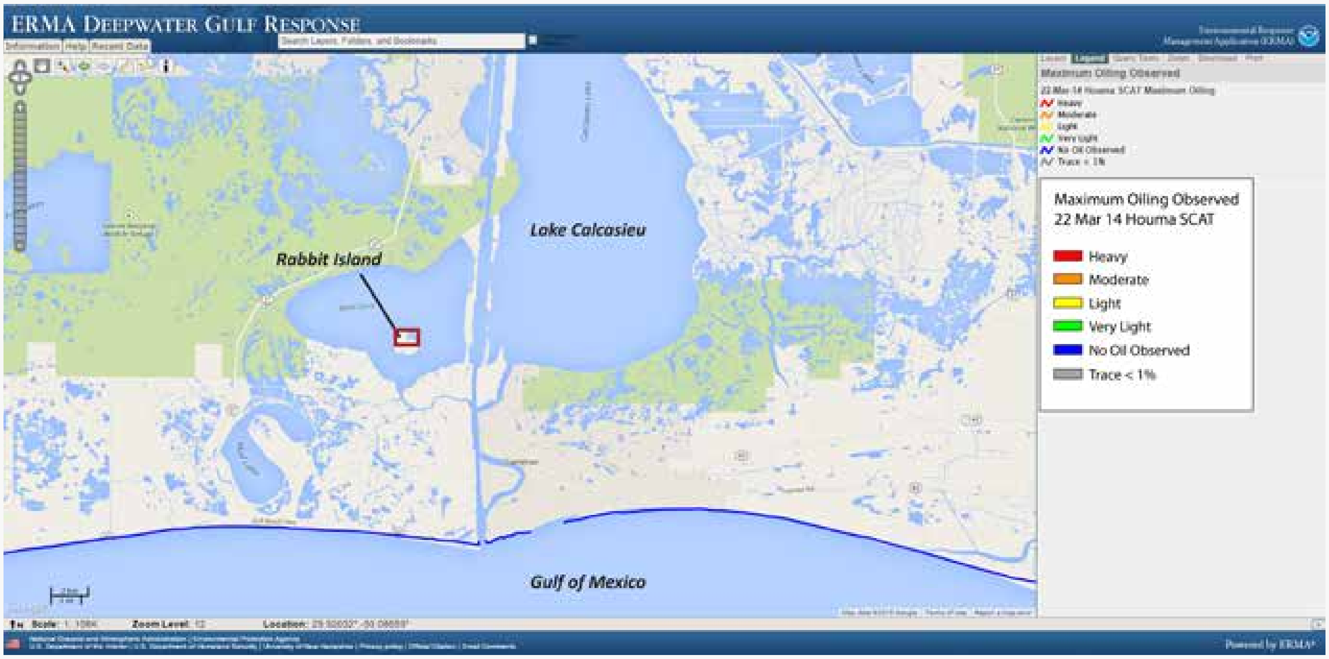

Figure S1.4. Modified screenshot from the ERMA Deepwater Gulf Response online mapping tool showing our sampling area (red box) in relation to the maximum shoreline oiling observed during Shoreline Cleanup Assessment Technique surveys. ERMA mapping tool: http://response.restoration.noaa.gov/maps-and-spatial-data/environmental-response-management-application-erma/erma-gulf-response.html (accessed 11/21/2016).

1. Rabbit Island (29°50’57.96”N, 93°22’51.53”W) Treatment: Control

Dermo prevalence reached levels high enough to be of concern in 2013, but there was little difference between average adult body size in the live population and the death assemblage baseline (Figs. S1.5, S1.6), suggesting the disease levels had little effect on oyster body size. There were also no trends in temperature or salinity that could have obscured effects of the DWH oil spill (Fig. S1.6).

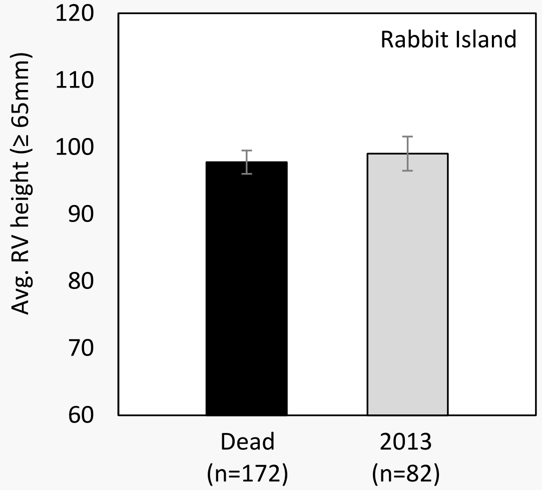

Figure S1.5. Average size of live oyster right valves (RV) ≥65 mm shell height in each year of sampling. Error bars represent the standard error of the mean.

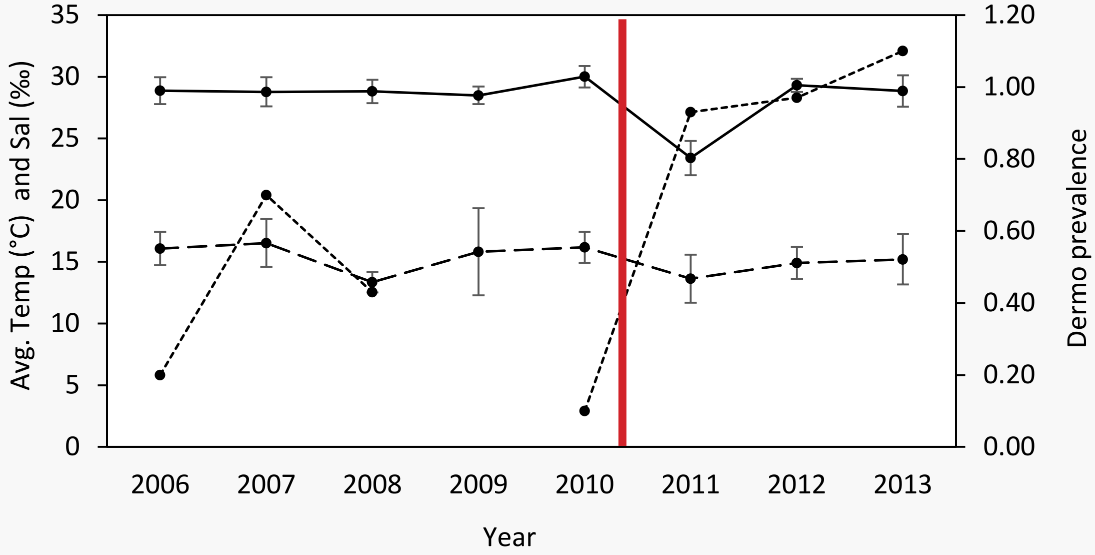

Figure S1.6. Trends in temperature (solid line), salinity (long-dashed line), and dermo prevalence (short-dashed line) through time at Coastwide Reference Monitoring System (CRMS) and Oyster Sentinel (OS) stations nearest our sampling sites. Error bars represent the standard error of the mean. Red bar indicates approximate timing of the DWH oil spill.

<u>Data sources</u>

Salinity & temperature data:	www.lacoast.gov/crms (CRMS 0685 - 29°53’23.28”N, 93°22’11.11”W; ~4.5 km north of Rabbit Island sampling sites)
Dermo prevalence data:	www.oystersentinel.org (N. E. Rabbit Island - 29°51’25.92”N, 93°21’47.08”W; ~1.75 km northeast of Rabbit Island sampling sites)

(a)

(b)

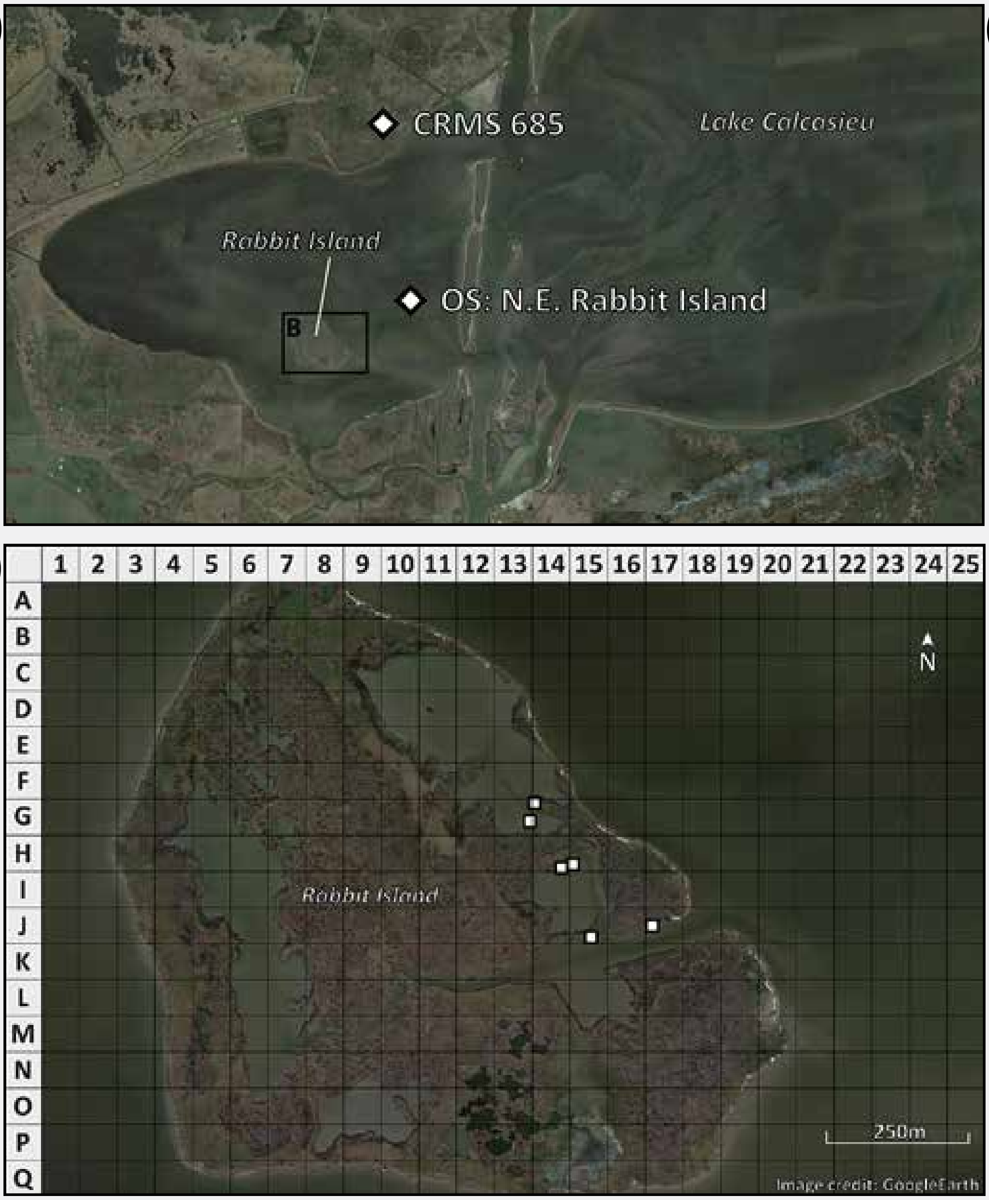

(c)

Year	Sample	Grid
2013	1	J15
	2	J17
	3	H14
	4	H15
	5	G14
	6	G13

Figure S1.7. Maps showing (a) the locations of Coastwide Reference Monitoring System (CRMS) and Oyster Sentinel (OS) stations closest to our sampling sites, and (b) a gridded satellite image showing the locations of sample sites for each year of sampling (squares = 2013). The reference table (c) lists the grid coordinates of each sample in panel (b). Satellite images are modified from Google Earth Pro version 7.1.2.2041.

Table S1.2. Table showing abundances of oysters in each sample. DA = death assemblage; LA = live assemblage.

Year	Sample	GPS coordinates	DA/LA	# specimens (≥65 mm)	# specimens (Total)
2013	1	29.848528, -93.380306	DA	30	141
			LA	12	55
2013	2	29.848694, -93.379194	DA	24	94
			LA	12	18
2013	3	29.849611, -93.380806	DA	20	59
			LA	10	15
2013	4	29.849667, -93.380611	DA	44	197
			LA	15	25
2013	5	29.850611, -93.381278	DA	18	81
			LA	10	10
2013	6	29.850333, -93.381341	DA	36	116
			LA	23	25

2. Marsh Island (29°35’35.59”N, 92° 0’35.47”W) Treatment: Control

Several oyster beds along Marsh Island in Vermilion Bay’s Southwest Pass, which connects the western portion of Vermilion Bay to the Gulf of Mexico (Fig. S1.8; Fig. S1.12), were sampled in 2011–2013. This locality was categorized as a control area because SCAT surveys observed no oil at these locations, although there was some oiling observed on Marsh Island further south, towards the Gulf of Mexico (Fig. S1.9). The locality was sampled three times for surface samples, in 2011, 2012, and 2013. Death assemblage samples were collected in 2011 (Table S1.3). Oyster right valves ≥65 mm in height were variable in abundance at Marsh Island, with live abundances across all sites varying from a low of nine in 2012 to 69 in 2013 (Table S1.4). The abundance of large oysters (≥65 mm in height) was higher in the death assemblages than in the live assemblages at this location, and there was little difference in the average heights between the live and death assemblages (Fig. S1.10).

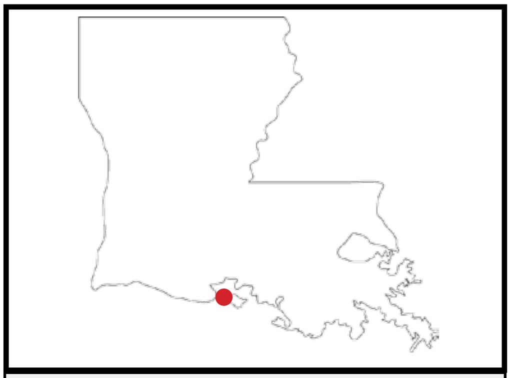

Figure S1.8. Map showing the location (red dot) of Marsh Island in Louisiana.

Environmental data from the nearest Coastwide Reference Monitoring System (CRMS) station to the sampling sites (CRMS 0541 - 29°36’58.32”N, 92°2’27.96”W; ~4 km northwest of our Marsh Island sampling sites; Fig. S1.12) showed that between 2008–2013, average water temperature ± se during the summer months, May–August, was 29°C ± 0.17. Average salinity ± se over the same interval was 8.8‰ ± 1.33. Data on the prevalence of *Perkinsus marinus* (dermo disease) infection from the nearest Oyster Sentinel station to our sampling sites (Indian Point - 29°37’6.6”N, 92°0’31.68”W; ~2.75 km north of our Marsh Island sampling sites; Fig. S1.12) indicated that average dermo prevalence ± se near Marsh Island was 0.03 ± 0.03 between 2010 and 2013. All environmental data and dermo prevalence data were accessed from www.lacoast.gov/crms and www.oystersentinel.org, respectively (Fig. S1.11).

Year	DA	LA		
	2011	2011	2012	2013
Samples	6	6	6	4

Table S1.3. Table showing the year(s) Marsh Island was sampled and the number of samples collected. DA = death assemblage; LA = live assemblage.

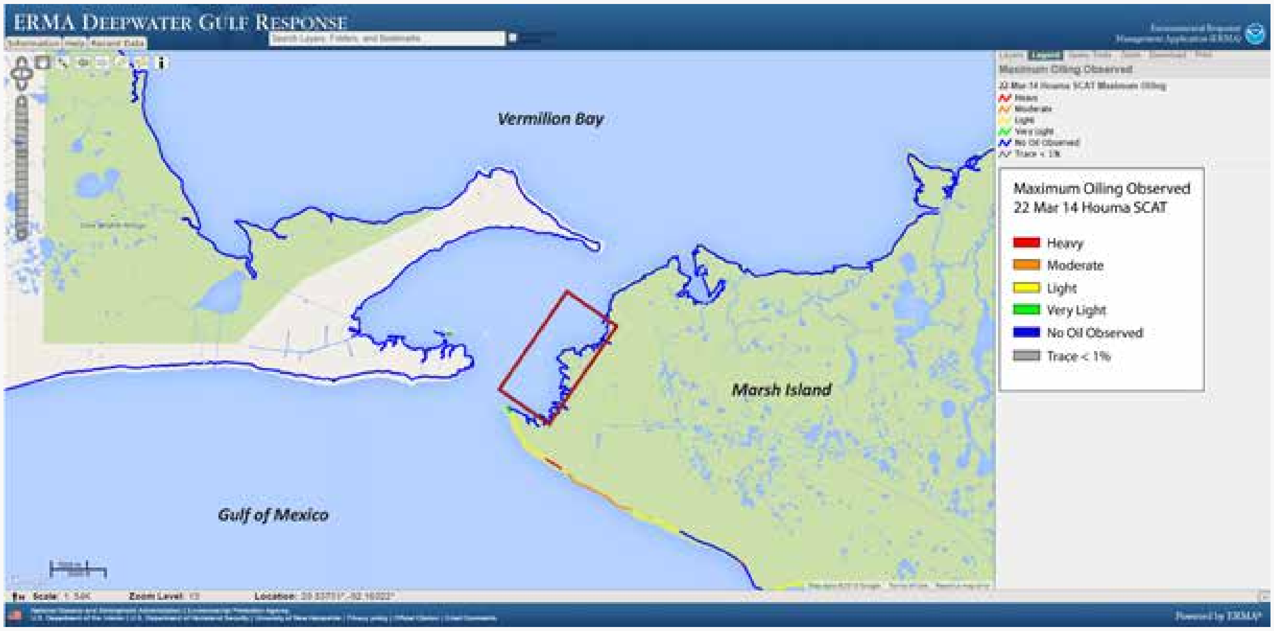

Figure S1.9. Modified screenshot from the ERMA Deepwater Gulf Response online mapping tool showing our sampling area (red box) in relation to the maximum shoreline oiling observed during Shoreline Cleanup Assessment Technique surveys. ERMA mapping tool: http://response.restoration.noaa.gov/maps-and-spatial-data/environmental-response-management-application-erma/erma-gulf-response.html (accessed 11/21/2016).

2. Marsh Island (29°35’35.59”N, 92° 0’35.47”W) Treatment: Control

Dermo prevalence was very low in 2010 and zero after 2010, suggesting that disease could not have affected the oyster body size results (Figs. S1.10, S1.11). The average salinity was low at CRMS 0541, but our sampling locations were closer to the Gulf of Mexico, so average salinity where the oyster beds were located may have been higher. The most important feature of these salinity data is that they do not show any patterns that could have obscured the impact of the DWH oil spill on oyster growth at this location. There were also no trends in temperature that could have caused oyster growth at this location to differ over time (Fig. S1.11).

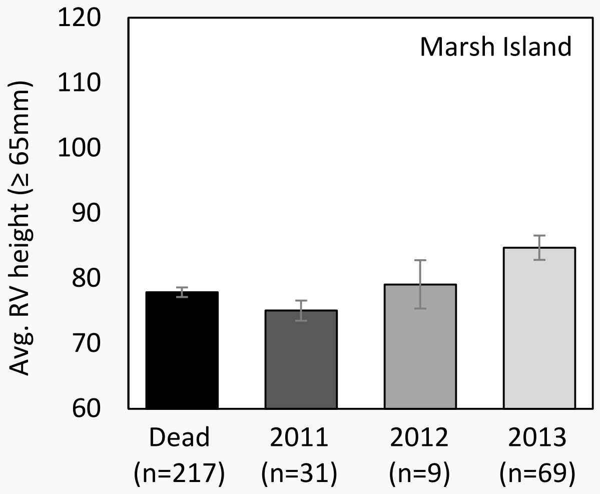

Figure S1.10. Average size of live oyster right valves (RV) ≥65 mm shell height in each year of sampling. Error bars represent the standard error of the mean.

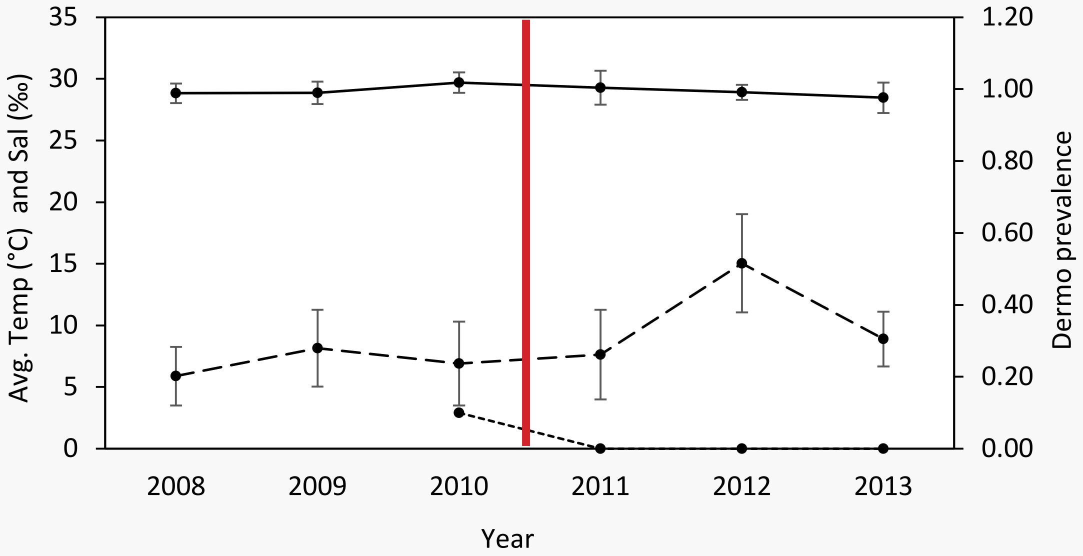

Figure S1.11. Trends in temperature (solid line), salinity (long-dashed line), and dermo prevalence (short-dashed line) through time at Coastwide Reference Monitoring System (CRMS) and Oyster Sentinel (OS) stations nearest our sampling sites. Error bars represent the standard error of the mean. Red bar indicates approximate timing of the DWH oil spill.

Data sources

Salinity & temperature data: www.lacoast.gov/crms (CRMS 0541 - 29°36’58.32”N, 92°2’27.96”W; ~4 km northwest of Marsh Island sampling sites)

Dermo prevalence data: www.oystersentinel.org (Indian Point - 29°37’6.6”N, 92°0’31.68”W; ~2.75 km north of Marsh Island sampling sites)

2. Marsh Island (29°35’35.59”N, 92° 0’35.47”W) Treatment: Control

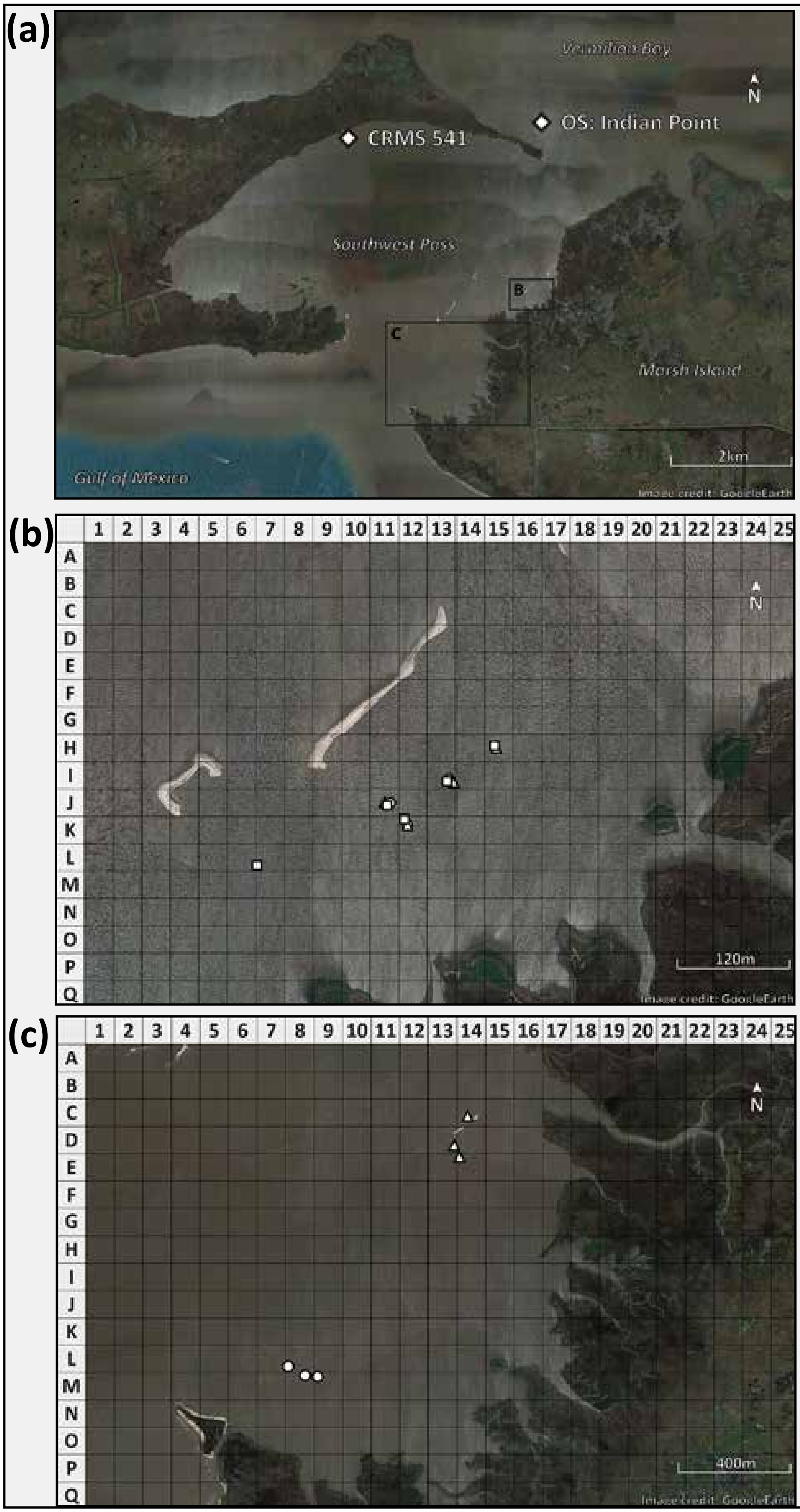

(d)

Year	Sample	Figure panel	Grid
2011 ○	1	B	J11
	2	B	I13
	3	B	K12
	4	C	M9
	5	C	M8
	6	C	L8
2012 △	1	B	H15
	2	B	I13
	3	B	K12
	4	B	J11
	5	C	D13
	6	C	C14
2013 □	1	B	H15
	2	B	I13
	3	B	K12
	4	B	J11

Figure S1.12. Maps showing (a) the locations of Coastwide Reference Monitoring System (CRMS) and Oyster Sentinel (OS) stations closest to our sampling sites, and (b) & (c) satellite images showing the locations of our sample sites in each year of sampling (circles = 2011, triangles = 2012, squares = 2013). The reference table (d) lists the grid coordinates of each sample in panels (b) and (c). Satellite images are modified from Google Earth Pro version 7.1.2.2041.

Table S1.4. Table showing abundances of oysters in each sample. DA = death assemblage; LA = live assemblage.

Year	Sample	GPS coordinates	DA/LA	# specimens (≥65 mm)	# specimens (Total)
2011	1	29.593261, -92.010819	DA	38	585
			LA	5	37
2011	2	29.593461, -92.01015	DA	35	1240
			LA	2	12
2011	3	29.593061, -92.010619	DA	43	623
			LA	4	28
2011	4	29.578217, -92.026767	DA	11	552
			LA	4	16
2011	5	29.578272, -92.02715	DA	41	415
			LA	8	26
2011	6	29.578525, -92.027811	DA	49	506
			LA	8	30
2012	1	29.593782, -92.009642	LA	2	9
2012	2	29.593461, -92.010118	LA	0	2
2012	3	29.593053, -92.010612	LA	0	1
2012	4	29.593264, -92.010863	LA	6	18
2012	5	29.585458, -92.021765	LA	1	14
2012	6	29.586397, -92.021264	LA	0	1
2013	1	29.593806, -92.009667	LA	20	36
2013	2	29.593472, -92.010167	LA	22	33
2013	3	29.593111, -92.010639	LA	10	22
2013	4	29.59325, -92.010861	LA	17	22

Several oyster beds along the southern edge of Caillou Lake (Fig. S1.13; Fig. S1.17) were sampled in 2011–2013. The oyster beds sampled occurred in a small bay and a nearby tidal channel. This locality was categorized as a control area because SCAT surveys observed no oil at these locations (Fig. S1.14). The locality was sampled three times for surface samples, in 2011, 2012, and 2013. Death assemblage samples were collected in 2011 (Table S1.5). Oyster right valves ≥65 mm in height were abundant in both the living and death assemblages at this location (Table S1.6), and there was very little difference in the average heights between the two assemblages (Fig. S1.15).

Environmental data from the nearest Coastwide Reference Monitoring System (CRMS) station to the sampling sites (CRMS 0383: 29°12’27.00”N, 90°56’56.04”W; ~1 km southwest of nearest Caillou Lake sampling sites) showed that between 2007–2013, average water temperature ± se during the summer months, May–August was 29.6°C ± 0.15 (Fig. S1.16). Average salinity ± se over the same interval was 12.2‰ ± 1.13 (Fig. S1.16). Data on the prevalence of *Perkinsus marinus* (dermo disease) infection from the nearest Oyster Sentinel station to our sampling sites (Old Camp - 29°12’58.32”N, 90°56’40.2”W; ~0.75 km northwest of nearest Caillou Lake sampling sites) indicated that average dermo prevalence ± se in Caillou Lake was 0.11 ± 0.04 between 2006 and 2013 (Fig. S1.16). All environmental and dermo prevalence data were accessed from www.lacoast.gov/crms and www.oystersentinel.org, respectively.

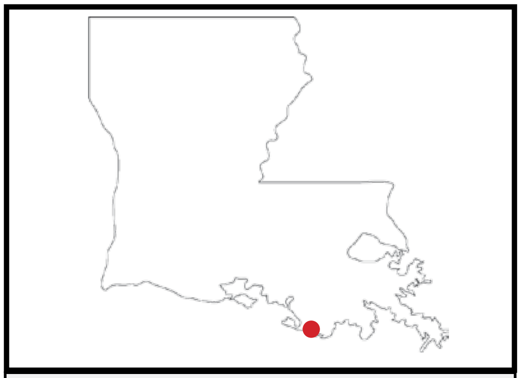

Figure S1.13. Map showing the location (red dot) of Caillou Lake in Louisiana.

Year	DA	LA		
	2011	2011	2012	2013
Samples	9	9	10	8

Table S1.5. Table showing the year(s) Caillou Lake was sampled and the number of samples collected. DA = death assemblage; LA = live assemblage.

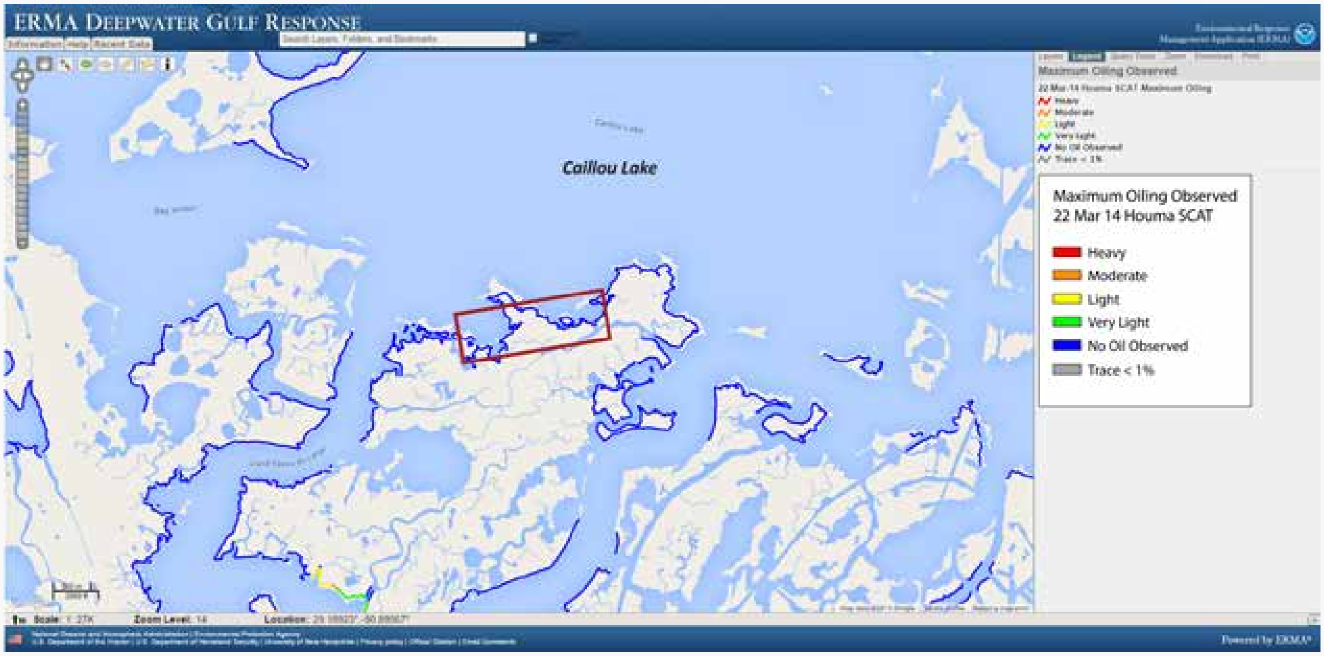

Figure S1.14. Modified screenshot from the ERMA Deepwater Gulf Response online mapping tool showing our sampling area (red box) in relation to the maximum shoreline oiling observed during Shoreline Cleanup Assessment Technique surveys. ERMA mapping tool: http://response.restoration.noaa.gov/maps-and-spatial-data/environmental-response-management-application-erma/erma-gulf-response.html (accessed 11/21/2016).

3. Caillou Lake (29°12’44.94”N, 90°55’47.06”W) Treatment: Control

There was an increase in average adult body size in the living population relative to the death assemblage baseline in 2012, but average body size decreased towards the baseline value again in 2013 (Fig. S1.15). There were no trends in temperature, salinity, or dermo prevalence that could have obscured the effects of the DWH oil spill on oyster growth at this location (Fig. S1.16).

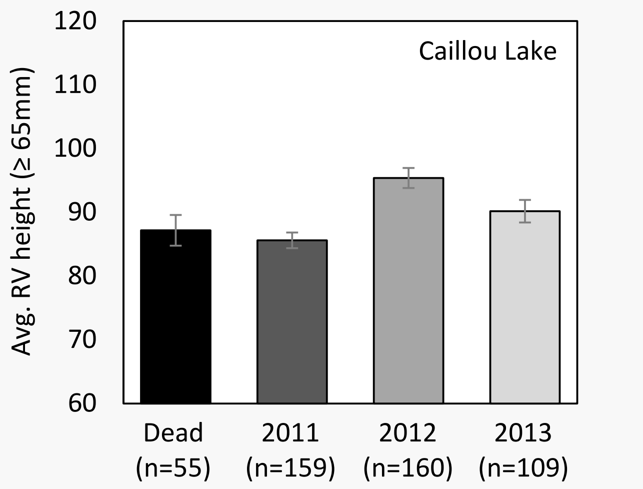

Figure S1.15. Average size of live oyster right valves (RV) ≥65 mm shell height in each year of sampling. Error bars represent the standard error of the mean.

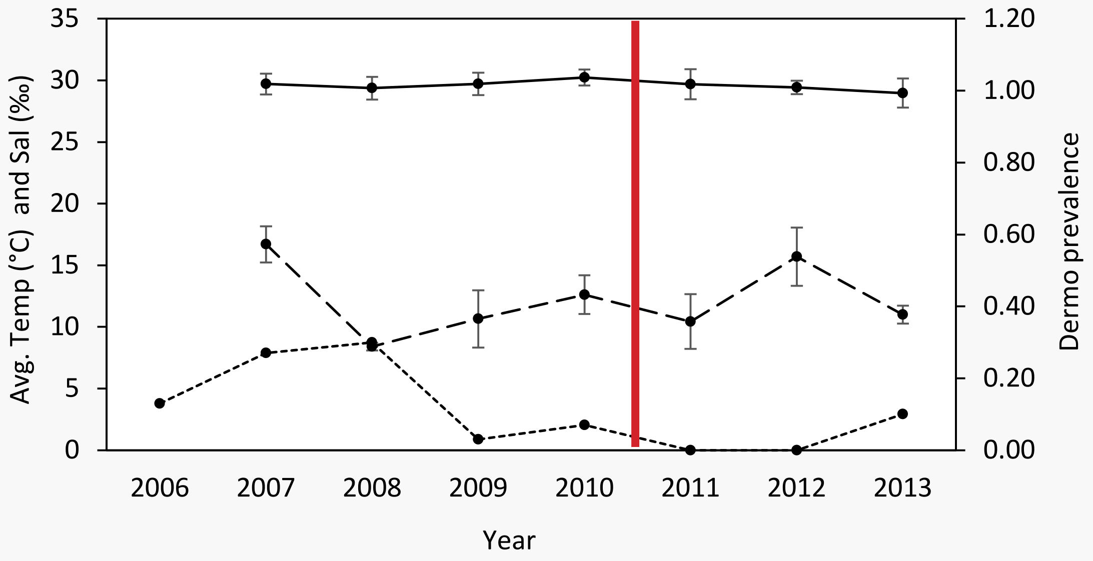

Figure S1.16. Trends in temperature (solid line), salinity (long-dashed line), and dermo prevalence (short-dashed line) through time at Coastwide Reference Monitoring System (CRMS) and Oyster Sentinel (OS) stations nearest our sampling sites. Error bars represent the standard error of the mean. Red bar indicates approximate timing of the DWH oil spill.

<u>Data sources</u>

Salinity & temperature data: www.lacoast.gov/crms (CRMS 0383 - 29°12’27.00”N, 90°56’56.04”W; ~1 km southwest of nearest Caillou Lake sampling sites)

Dermo prevalence data: www.oystersentinel.org (Old Camp - 29°12’58.32”N, 90°56’40.2”W; ~0.75 km northwest of nearest Caillou Lake sampling sites)

3. Caillou Lake (29°12’44.94”N, 90°55’47.06”W) — Treatment: Control

(a) (b) (c)

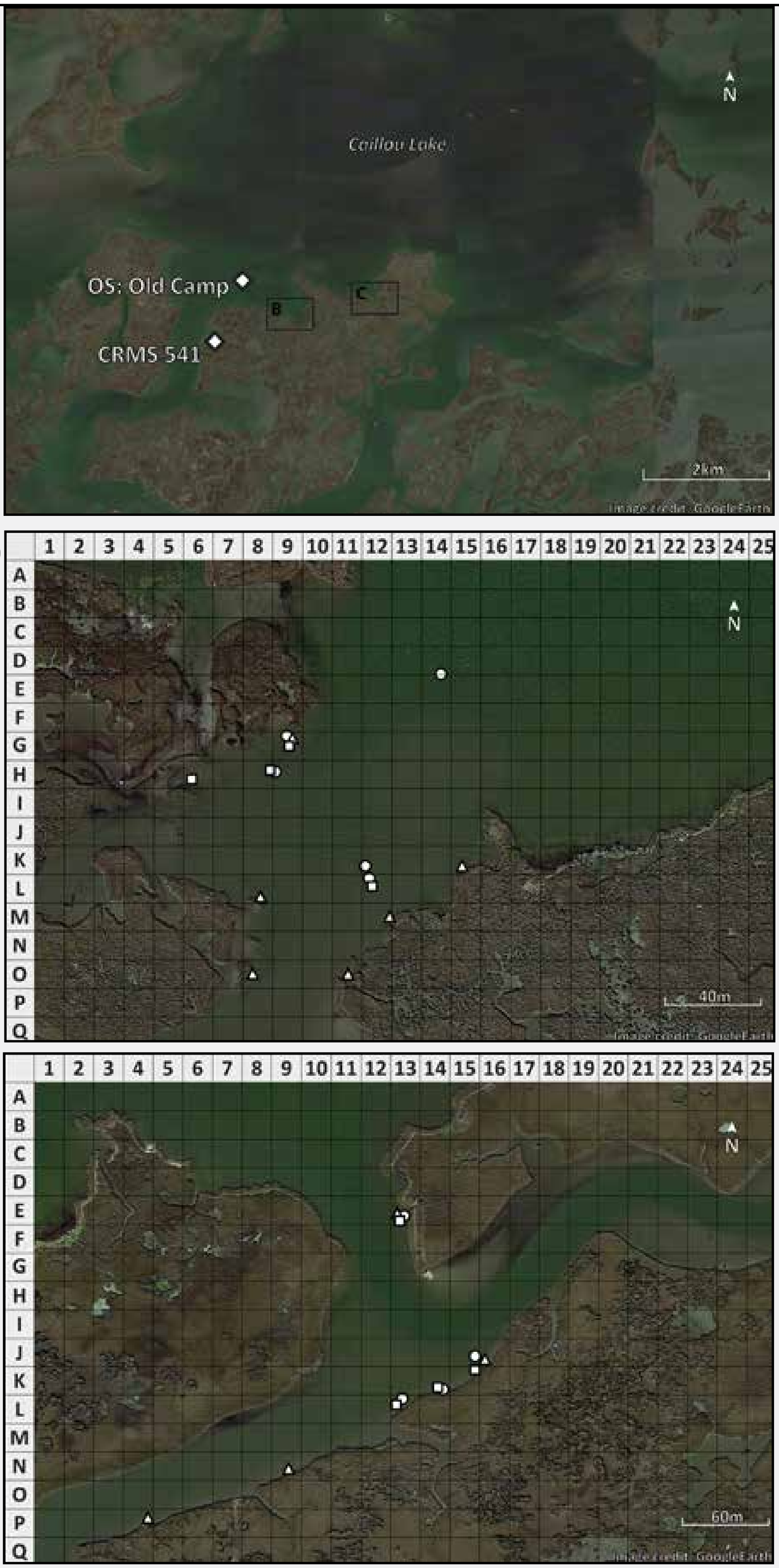

(d)

Year	Sample	Figure panel	Grid
2011 ○	1	B	G9
	2	B	H9
	3	C	E13
	4	B	D14
	5	B	L12
	6	B	K12
	7	C	J15
	8	C	L13
	9	C	K14
2012 △	1	B	K15
	2	B	M12
	3	B	O11
	4	B	O8
	5	B	L8
	6	B	G9
	7	C	J16
	8	C	N9
	9	C	P4
	10	C	E13
2013 □	1	B	H8
	2	B	G9
	3	B	H6
	4	C	E13
	5	C	L13
	6	C	K14
	7	C	K15
	8	B	L12

Figure S1.17. Maps showing (a) the locations of Coastwide Reference Monitoring System (CRMS) and Oyster Sentinel (OS) stations closest to our sampling sites, and (b) & (c) satellite images showing the specific locations of the sample sites in each year of sampling (circles = 2011, triangles = 2012, squares = 2013). The reference table (d) lists the grid coordinates of each sample in panels (b) and (c). Satellite images are modified from Google Earth Pro version 7.1.2.2041.

Table S1.6. Table showing abundances of oysters in each sample. DA = death assemblage; LA = live assemblage.

Year	Sample	GPS coordinates	DA/LA	# specimens (≥65 mm)	# specimens (Total)
2011	1	29.211153, -90.939064	DA	3	208
			LA	5	35
2011	2	29.211019, -90.939119	DA	13	185
			LA	19	32
2011	3	29.213389, -90.923389	DA	0	83
			LA	16	61
2011	4	29.211358, -90.938403	DA	0	34
			LA	29	78
2011	5	29.210617, -90.938731	DA	3	189
			LA	15	46
2011	6	29.210661, -90.93875	DA	1	154
			LA	18	33
2011	7	29.21265, -90.923	DA	14	292
			LA	32	55
2011	8	29.212444, -90.923433	DA	4	38
			LA	10	64
2011	9	29.212483, -90.923192	DA	17	354
			LA	15	41
2012	1	29.210646, -90.938332	LA	16	48
2012	2	29.210472, -90.938665	LA	18	64
2012	3	29.210277, -90.938851	LA	14	31
2012	4	29.210265, -90.939224	LA	10	14
2012	5	29.210545, -90.939163	LA	9	36
2012	6	29.211134, -90.939007	LA	13	48
2012	7	29.212613, -90.922968	LA	10	12
2012	8	29.212111, -90.924105	LA	20	34
2012	9	29.211872, -90.924933	LA	9	24
2012	10	29.213389, -90.923414	LA	41	110
2013	1	29.211028, -90.939139	LA	13	22
2013	2	29.211111, -90.939056	LA	11	24
2013	3	29.211, -90.939472	LA	9	29
2013	4	29.213361, -90.923417	LA	13	28
2013	5	29.212417, -90.923472	LA	14	31
2013	6	29.2125, -90.923222	LA	14	57
2013	7	29.212583, -90.923	LA	19	32
2013	8	29.210583, -90.938722	LA	16	48

4. LUMCON (29°15’16.27”N, 90°39’51.09”W) Treatment: Control

Several oyster beds in marsh channels adjacent to the Louisiana Universities Marine Consortium (LUMCON) located in Cocodrie, LA (Figs. S1.18, S1.20) were sampled in 2011–2013. The oyster beds sampled were fringing beds along the edges of marsh tidal channels. This locality was categorized as a control area because SCAT surveys observed no oil at this location (Fig. S1.22). The locality was sampled three times for surface samples, in 2011, 2012, and 2013. Death assemblage samples were collected in 2011 (Table S1.7). Oyster right valves ≥65 mm in height were abundant in both the living and death assemblages at this location (Table S1.8), and average body sizes increased in 2012 and stayed above the death assemblage baseline in 2013 (Fig. S1.19).

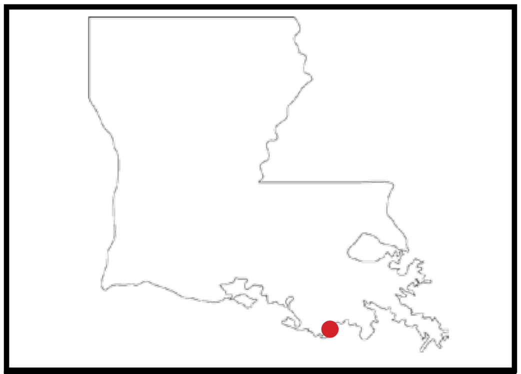

Figure S1.18. Map showing the location (red dot) of LUMCON in Louisiana.

Environmental data from the nearest Coastwide Reference Monitoring System (CRMS) station to the sampling sites (CRMS 0369: 29°17’40.56”N, 90°41’52.8”W; ~5 km northwest of nearest LUMCON sampling sites) showed that between 2007–2013, average water temperature ± se during the summer months, May–August, was 29.0°C ± 0.32 (Fig. S1.21). Average salinity ± se over the same interval was 4.7‰ ± 0.93 (Fig. S1.21). Data on the prevalence of *Perkinsus marinus* (dermo disease) infection from the nearest Oyster Sentinel station to our sampling sites (Bay Tambour - 29°11’14.64”N, 90°39’55.44”W; ~7.5 km south of nearest LUMCON sampling sites) indicated that average dermo prevalence ± se near LUMCON was 0.3 ± 0.08 between 2000 and 2009 (Fig. S1.21). We were unable to find more recent dermo prevalence data from this location. All environmental and dermo prevalence data were accessed from www.lacoast.gov/crms and www.oystersentinel.org, respectively.

Year	DA	LA		
	2011	2011	2012	2013
Samples	9	9	6	9

Table S1.7. Table showing the year(s) LUMCON was sampled and the number of samples collected. DA = death assemblage; LA = live assemblage.

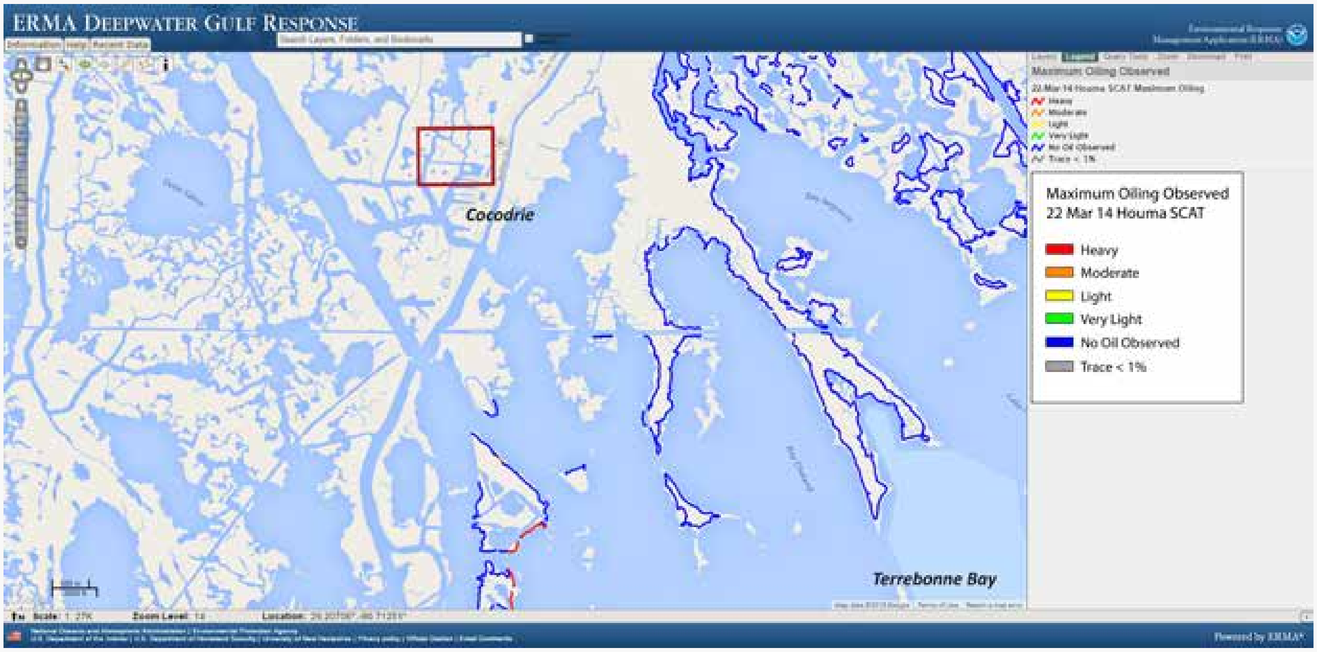

Figure S1.19. Modified screenshot from the ERMA Deepwater Gulf Response online mapping tool showing our sampling area (red box) in relation to the maximum shoreline oiling observed during Shoreline Cleanup Assessment Technique surveys. ERMA mapping tool: http://response.restoration.noaa.gov/maps-and-spatial-data/environmental-response-management-application-erma/erma-gulf-response.html (accessed 11/21/2016).

Note that the salinity at our sampling locations was almost certainly higher than the salinity at CRMS 0369, given its location further from the Gulf of Mexico. There were no patterns in temperature, salinity, or dermo prevalence that could have caused trends in average oyster body size that would have masked effects of the DWH oil spill (Fig. S1.21).

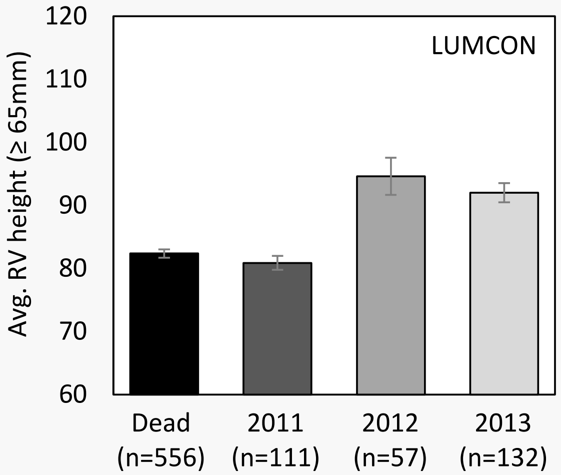

Figure S1.20. Average size of live oyster right valves (RV) ≥65 mm shell height in each year of sampling. Error bars represent the standard error of the mean.

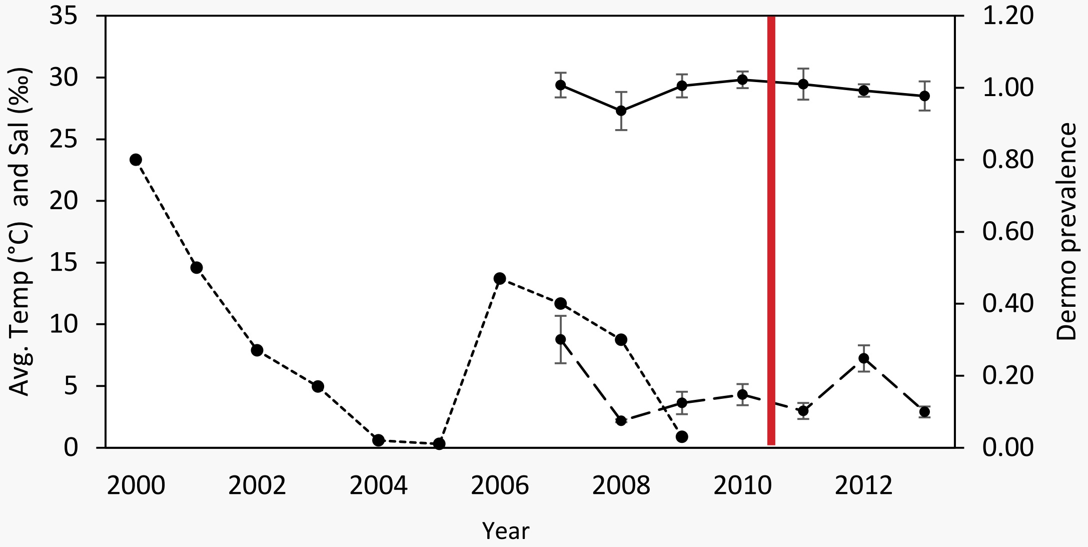

Figure S1.21. Trends in temperature (solid line), salinity (long-dashed line), and dermo prevalence (short-dashed line) through time at Coastwide Reference Monitoring System (CRMS) and Oyster Sentinel (OS) stations nearest our sampling sites. Error bars represent the standard error of the mean. Red bar indicates approximate timing of the DWH oil spill.

Data sources

Salinity & temperature data: www.lacoast.gov/crms (CRMS 0369 - 29°17’40.56”N, 90°41’52.8”W; ~5 km northwest of nearest LUMCON sampling sites)

Dermo prevalence data: www.oystersentinel.org (Bay Tambour - 29°11’14.64”N, 90°39’55.44”W; ~7.5 km south of nearest LUMCON sampling sites)

(a)

(b)

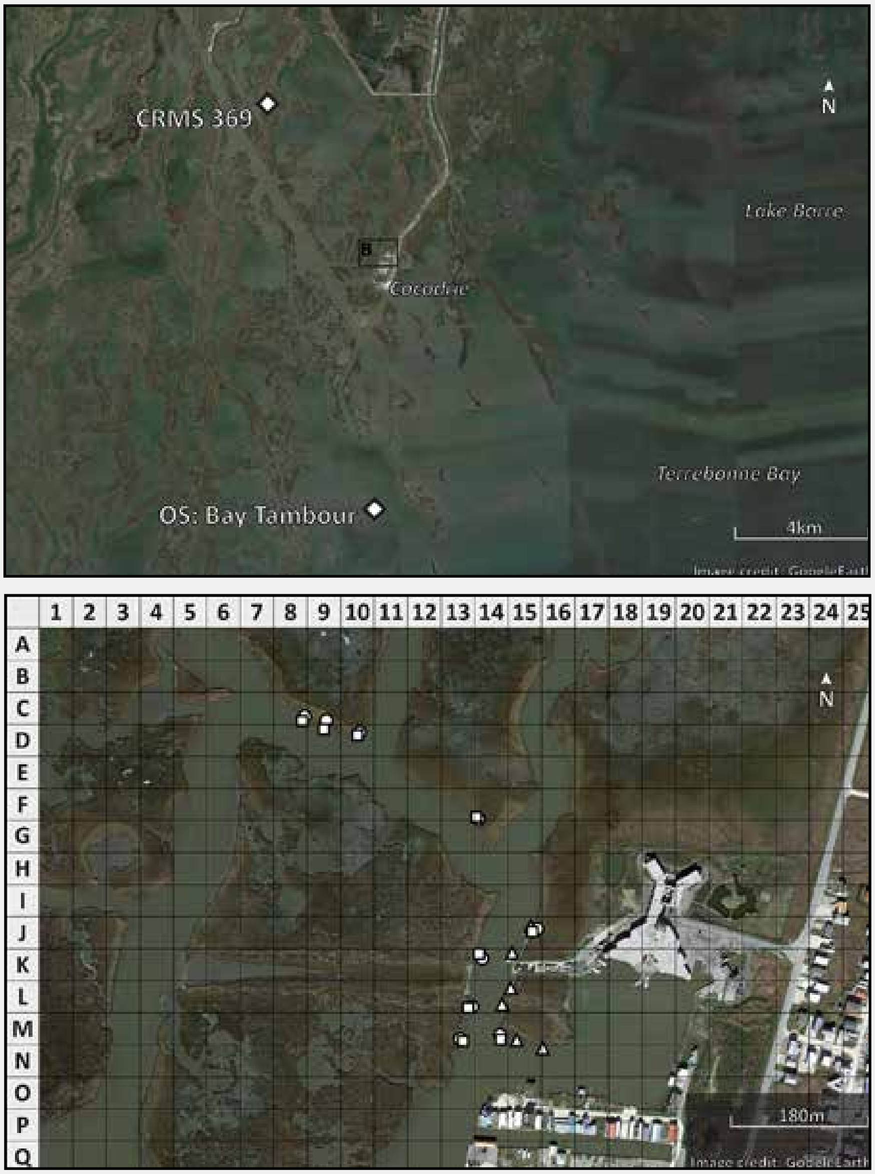

(c)

Year	Sample	Grid
2011 ○	1	M14
	2	M13
	3	L13
	4	K14
	5	J15
	6	D10
	7	F14
	8	C8
	9	C9
2012 △	1	J15
	2	L14
	3	K15
	4	L15
	5	M15
	6	N16
2013 □	1	C8
	2	D10
	3	D9
	4	F14
	5	J15
	6	K14
	7	L13
	8	M13
	9	M14

Figure S1.22. Maps showing (a) the locations of Coastwide Reference Monitoring System (CRMS) and Oyster Sentinel (OS) stations closest to our sampling sites, and (b) a gridded satellite image showing the locations of the sample sites in each year of sampling (circles = 2011, triangles = 2012, squares = 2013). The reference table (c) lists the grid coordinates of each sample in panel (b). Satellite images are modified from Google Earth Pro version 7.1.2.2041.

Table S1.8. Table showing abundances of oysters in each sample. DA = death assemblage; LA = live assemblage.

Year	Sample	GPS coordinates	DA/LA	# specimens (≥65 mm)	# specimens (Total)
2011	1	29.253086, -90.6639	DA	29	718
			LA	13	33
2011	2	29.253033, -90.664389	DA	63	1526
			LA	4	30
2011	3	29.253414, -90.664267	DA	69	1000
			LA	6	19
2011	4	29.253978, -90.664133	DA	31	495
			LA	7	28
2011	5	29.254314, -90.663394	DA	13	171
			LA	7	17
2011	6	29.256583, -90.665742	DA	97	688
			LA	18	29
2011	7	29.255594, -90.664214	DA	110	582
			LA	13	21
2011	8	29.256789, -90.666464	DA	86	1132
			LA	28	38
2011	9	29.256733, -90.666183	DA	58	563
			LA	15	31
2012	1	29.254303, -90.663482	LA	18	26
2012	2	29.253445, -90.663862	LA	14	36
2012	3	29.254035, -90.663721	LA	7	19
2012	4	29.25363, -90.663774	LA	3	4
2012	5	29.253015, -90.663694	LA	9	18
2012	6	29.252944, -90.663337	LA	6	9
2013	1	29.25675, -90.6665	LA	20	53
2013	2	29.256556, -90.665778	LA	14	50
2013	3	29.256639, -90.666222	LA	10	62
2013	4	29.255583, -90.664222	LA	19	45
2013	5	29.254306, -90.663472	LA	10	13
2013	6	29.254028, -90.664167	LA	15	34
2013	7	29.253389, -90.664306	LA	9	29
2013	8	29.253, -90.664389	LA	20	48
2013	9	29.253028, -90.663889	LA	15	34

Several oyster beds in marsh pools adjacent to Bay Bourboux in northern Terrebonne Bay (Figs. S1.23, S1.27) were sampled in 2013. The oyster beds sampled occurred in marsh tidal channels and formed pillar-shaped clumps stuck deeply into the mud. This locality was categorized as an impact area because SCAT surveys observed moderate oiling along the adjacent marsh edge (Fig. S1.24). Collections were made at the locality for live and death assemblage samples once, in 2013 (Table S1.9). Six samples were collected. Oyster right valves ≥65 mm in height were abundant in both the living and death assemblages at this location (Table S1.10), and there was very little difference in the average heights between the two assemblages (Fig. S1.25).

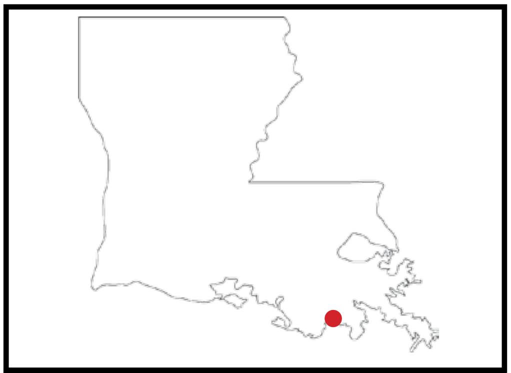

Figure S1.23. Map showing the location (red dot) of Bay Bourbeux in Louisiana.

Environmental data from the nearest Coastwide Reference Monitoring System (CRMS) station to the sampling sites (CRMS 0341: 29°18’32.76”N, 90°30’44.28”W; ~4.5 km east of nearest Bay Bourbeux sampling sites) showed that between 2007–2013, average water temperature ± se during the summer months, May–August, was 29.2°C ± 0.18 (Fig. S1.26). Average salinity ± se over the same interval was 16.3‰ ± 1.05 (Fig. S1.26). Data on the prevalence of *Perkinsus marinus* (dermo disease) infection, averaged from the two nearest Oyster Sentinel stations to our sampling sites (Lake Felicity: 29°18’58.68”N, 90°26’48.48”W, and Lake Chien: 29°20’3.12”N, 90°26’51.36”W; Both ~10.75 km east of nearest Bay Bourbeux sampling sites) indicated that average dermo prevalence ± se near Bay Bourbeux was 0.18 ± 0.06 between 2007 and 2013 (Fig. S1.26). All environmental and dermo prevalence data were accessed from www.lacoast.gov/crms and www.oystersentinel.org, respectively.

Year	DA	LA		
	2011	2011	2012	2013
Samples	6	N/A	N/A	6

Table S1.9. Table showing the year(s) Bay Bourbeux was sampled and the number of samples collected. DA = death assemblage; LA = live assemblage.

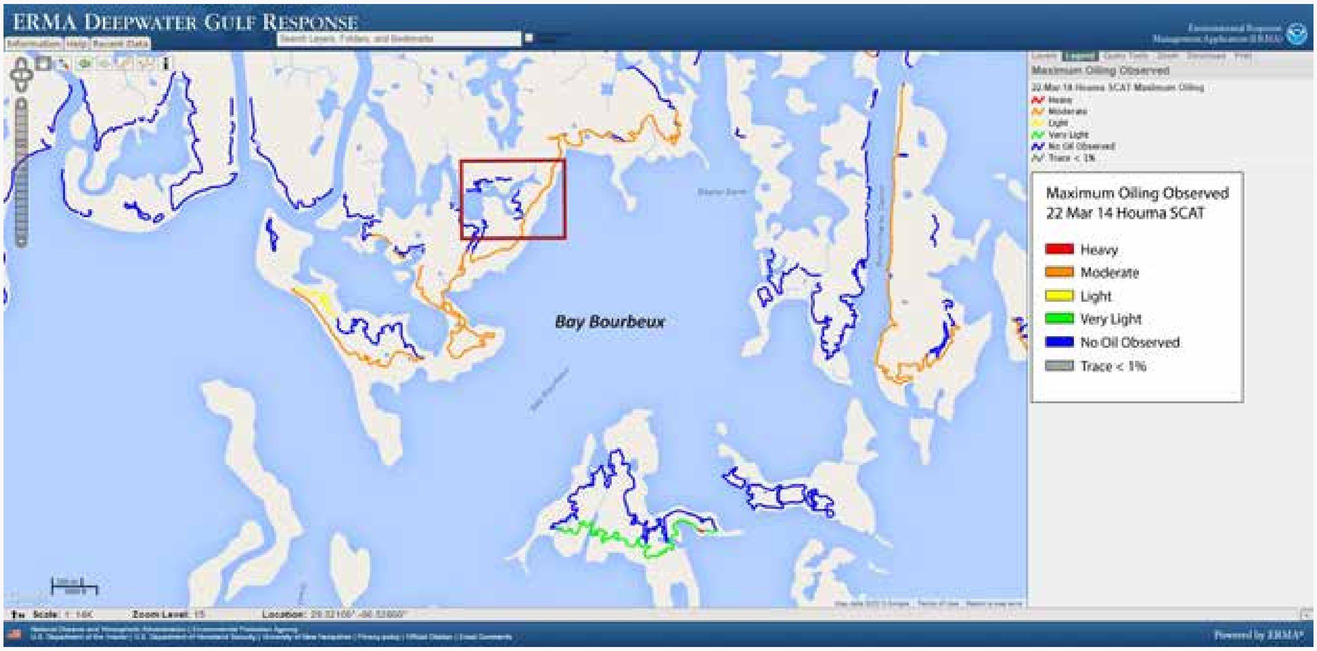

Figure S1.24. Modified screenshot from the ERMA Deepwater Gulf Response online mapping tool showing our sampling area (red box) in relation to the maximum shoreline oiling observed during Shoreline Cleanup Assessment Technique surveys. ERMA mapping tool: http://response.restoration.noaa.gov/maps-and-spatial-data/environmental-response-management-application-erma/erma-gulf-response.html (accessed 11/21/2016).

Although dermo prevalence rose between 2011 and 2013, it never reached levels that would be expected to cause oyster mortality. Further, the average body size of living oysters in 2013 was similar to the death assemblage baseline (Figs. S1.25, S1.26), suggesting there was no effect of disease that could have obscured or resembled impact from the DWH oil spill. Likewise, there were no trends in temperature or salinity that could have obscured effects of the DWH oil spill (Fig. S1.26).

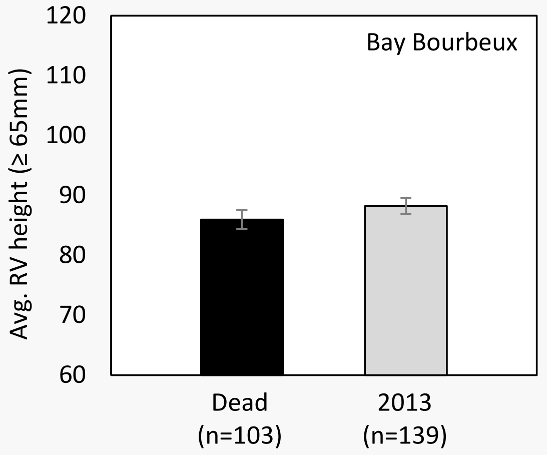

Figure S1.25. Average size of live oyster right valves (RV) ≥65 mm shell height in each year of sampling. Error bars represent the standard error of the mean.

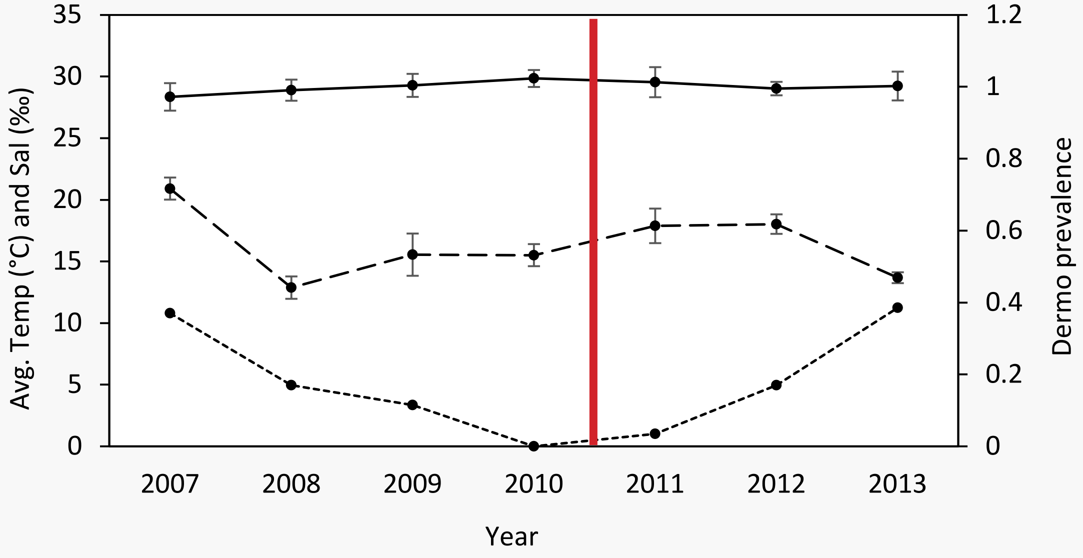

Figure S1.26. Trends in temperature (solid line), salinity (long-dashed line), and dermo prevalence (short-dashed line) through time at Coastwide Reference Monitoring System (CRMS) and Oyster Sentinel (OS) stations nearest our sampling sites. Error bars represent the standard error of the mean. Red bar indicates approximate timing of the DWH oil spill.

Data sources

Salinity & temperature data: www.lacoast.gov/crms (CRMS 0341 - 29°18’32.76”N, 90°30’44.28”W; ~4.5 km east of nearest Bay Bourbeux sampling sites)

Dermo prevalence data: www.oystersentinel.org (Averaged data from Lake Felicity - 29°18’58.68”N, 90°26’48.48”W, and Lake Chien - 29°20’3.12”N, 90°26’51.36”W; Both ~10.75 km east of nearest Bay Bourbeux sampling sites)

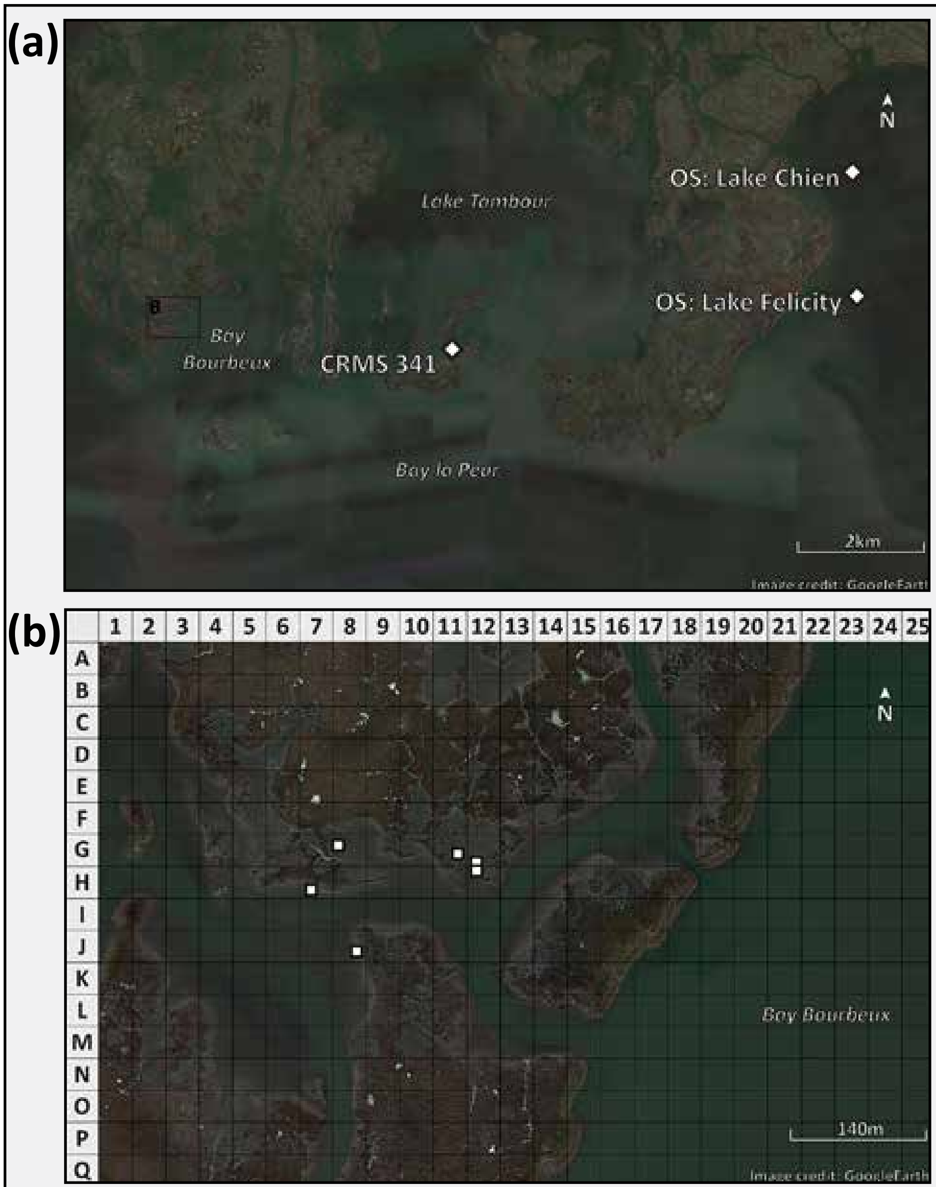

(c)

Year	Sample	Grid
2013	1	G11
□	2	H12
	3	G12
	4	G8
	5	J8
	6	H7

Figure S1.27. Maps showing (a) the locations of Coastwide Reference Monitoring System (CRMS) and Oyster Sentinel (OS) stations closest to our sampling sites, and (b) a gridded satellite image showing the locations of the sample sites in each year of sampling (squares = 2013). The reference table (c) lists the grid coordinates of each sample in panel (b). Satellite images are modified from Google Earth Pro version 7.1.2.2041.

Table S1.10. Table showing abundances of oysters in each sample. DA = death assemblage; LA = live assemblage.

Year	Sample	GPS coordinates	DA/LA	# specimens (≥65 mm)	# specimens (Total)
2013	1	29.314194, -90.557639	DA	8	48
			LA	16	37
2013	2	29.314056, -90.557444	DA	13	137
			LA	21	41
2013	3	29.314111, -90.557444	DA	18	339
			LA	32	73
2013	4	29.314278, -90.558889	DA	17	81
			LA	27	42
2013	5	29.313306, -90.558694	DA	27	413
			LA	23	79
2013	6	29.313861, -90.559167	DA	20	132
			LA	20	45

Several oyster beds on marsh flats along the southeastern edge of Mendicant Island in southern Barataria Bay (Figs. S1.28, S1.30) were sampled in 2011–2013. The oyster beds sampled occurred along the edges of mudflats ringed by marsh grass. This locality was categorized as an impact area because SCAT surveys observed moderate oiling adjacent to this location (Fig. S1.32). The locality was sampled three times for surface samples, in 2011, 2012, and 2013. Death assemblage samples were collected in 2011 (Table S1.11). Oyster right valves ≥65 mm in height were abundant in both the living and death assemblages at this location (Table S1.12). There was little difference in the average heights between the two assemblages, although, as was the case at other localities, there was a slight increase in average sizes from 2011 to 2012 that persisted into 2013 (Fig. S1.29).

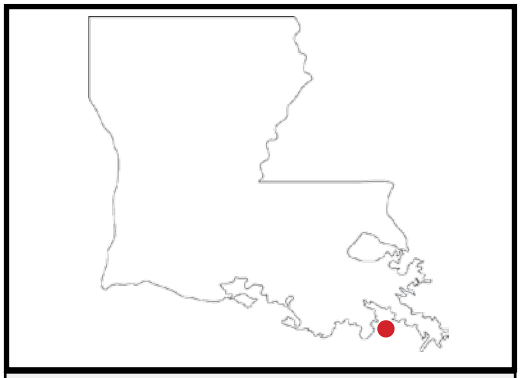

Figure S1.28. Map showing the location (red dot) of Mendicant Island in Louisiana.

Environmental data from the two nearest Coastwide Reference Monitoring System (CRMS) stations to all of our Barataria Bay sampling sites (CRMS 0171 - 29°19’25.68”N, 89°47’45.6”W; ~17.25 km east of nearest Mendicant Island sampling sites and CRMS 0178 - 29°17’15.00”N, 90°2’49.20”W; ~7.25 km southwest of nearest Mendicant Island sampling sites) showed that between 2007–2013, average water temperature ± se during the summer months, May–August, was 29.0°C ± 0.18 and 29.04°C ± 0.17 at CRMS 0171 and 0178, respectively (Fig. S1.31). Average salinity ± se over the same interval was 14.4‰ ± 1.57 at CRMS 0171 and 16.1‰ ± 1.17 at CRMS 0178 (Fig. S1.31). Data on the prevalence of *Perkinsus marinus* (dermo disease) infection from the nearest Oyster Sentinel station to the sampling sites (Middle Hackberry Bay - 29°24’6.12”N, 90°1’48”W; ~11.75 km northwest of nearest Mendicant Island sampling sites) indicated that average dermo prevalence ± se in southern Barataria Bay was 0.28 ± 0.12 between 2005 and 2013 (although no data were available for 2011 or 2012; Fig. S1.31).

Year	DA	LA		
	2011	2011	2012	2013
Samples	9	9	7	9

Table S1.11. Table showing the year(s) Mendicant Island was sampled and the number of samples collected. DA = death assemblage; LA = live assemblage.

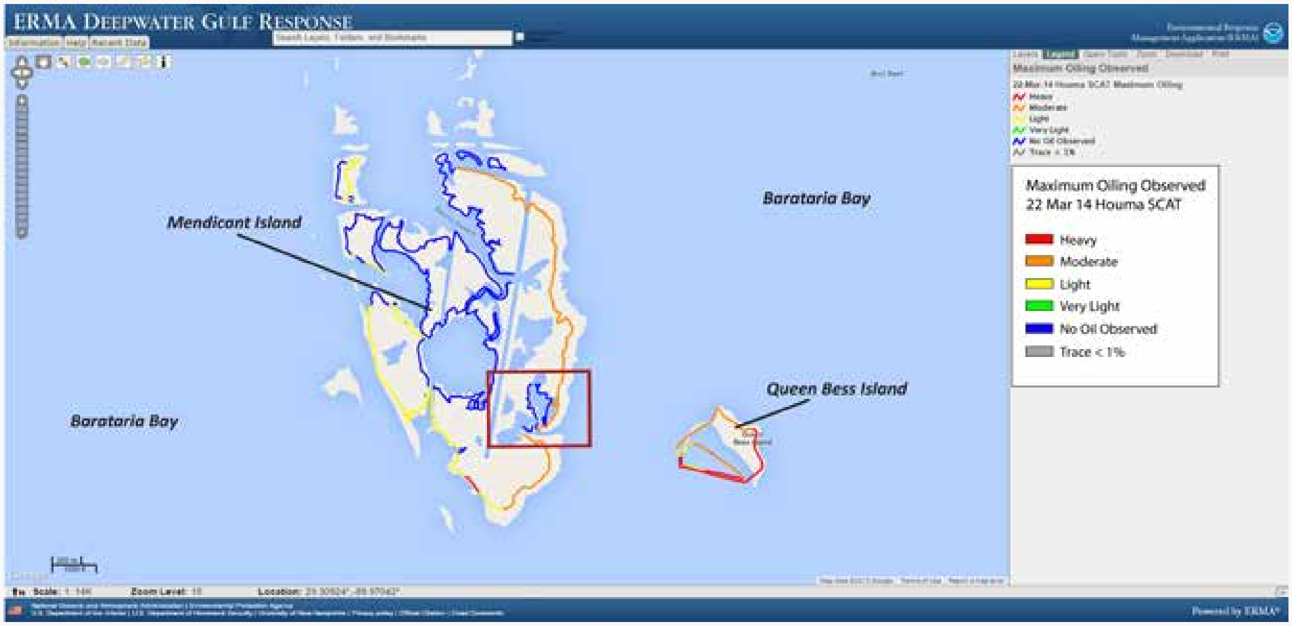

Figure S1.29. Modified screenshot from the ERMA Deepwater Gulf Response online mapping tool showing our sampling area (red box) in relation to the maximum shoreline oiling observed during Shoreline Cleanup Assessment Technique surveys. ERMA mapping tool: http://response.restoration.noaa.gov/maps-and-spatial-data/environmental-response-management-application-erma/erma-gulf-response.html (accessed 11/21/2016).

All environmental data and dermo prevalence data were accessed from www.lacoast.gov/crms and www.oystersentinel.org, respectively.

Average oyster body size increased in 2012 and 2013 above the pre-spill baseline (Fig. S1.30), opposite the expected pattern if the DWH oil spill had negatively affected oyster growth. Salinity increased in 2012, which may have been beneficial for oyster growth, but had there been a serious impact of the DWH oil spill, the modest shift in salinity would have been unlikely to counteract its negative effects. There were no trends in temperature that could have obscured the DWH oil spill’s effects on oyster body size. Likewise, dermo prevalence was below levels expected to cause oyster mortality in all years for which data were available, so could not have masked effects of the spill (Fig. S1.31).

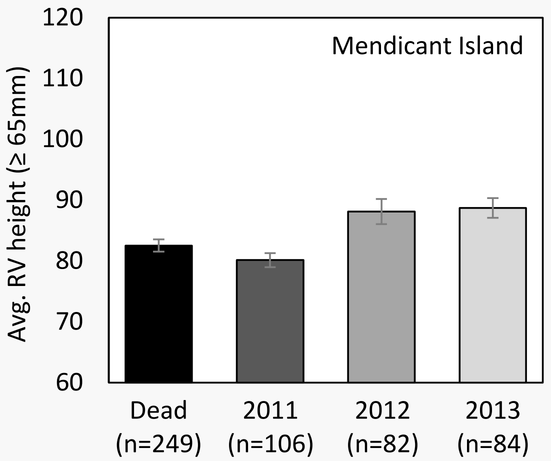

Figure S1.30. Average size of live oyster right valves (RV) ≥65 mm shell height in each year of sampling. Error bars represent the standard error of the mean.

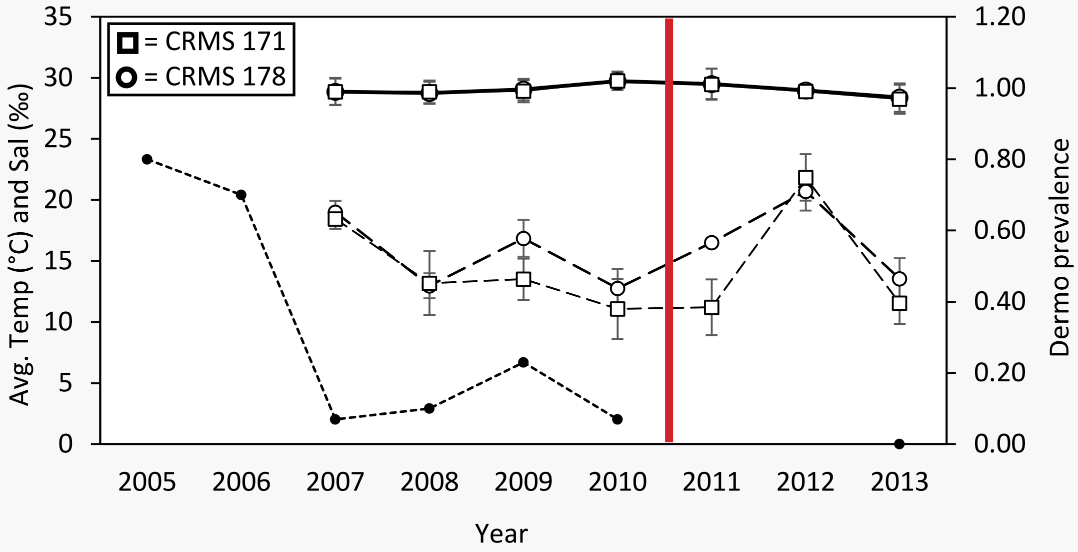

Figure S1.31. Trends in temperature (solid lines), salinity (long-dashed lines), and dermo prevalence (short-dashed line) through time at Coastwide Reference Monitoring System (CRMS; white points) and Oyster Sentinel (OS; black points) stations nearest our sampling sites. Error bars represent the standard error of the mean. Red bar indicates approximate timing of the DWH oil spill.

Data sources

Salinity & temperature data: www.lacoast.gov/crms

A) CRMS 0171 - 29°19’25.68”N, 89°47’45.6”W; ~17.25 km east of nearest Mendicant Island sampling sites

B) CRMS 0178 - 29°17’15.00”N, 90°2’49.20”W; ~7.25 km southwest of nearest Mendicant Island sampling sites

Dermo prevalence data: www.oystersentinel.org (Middle Hackberry Bay - 29°24’6.12”N, 90°1’48”W; ~11.75 km northwest of nearest Mendicant Island sampling sites)

(a)

(b)

(c)

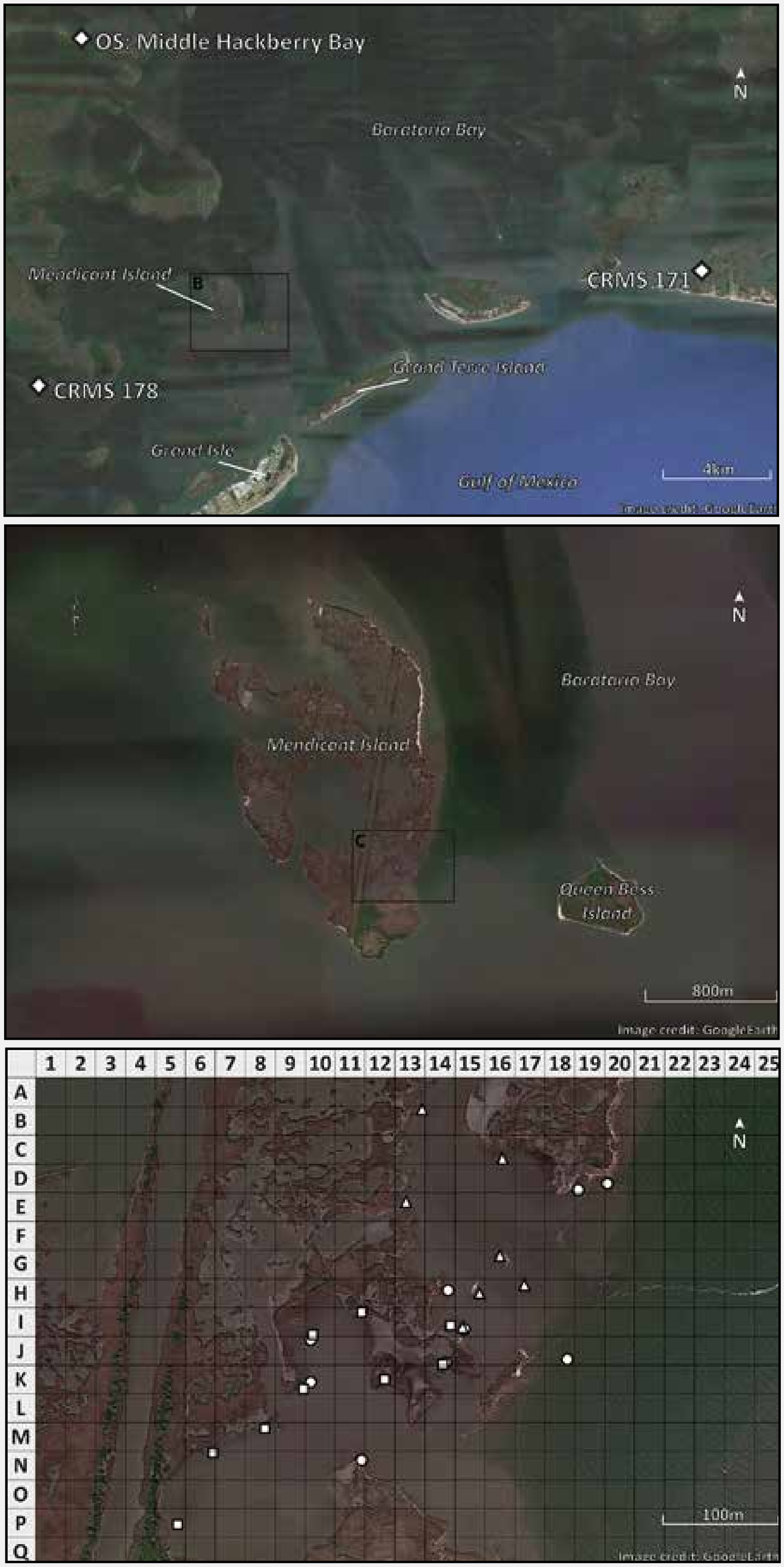

(d)

Year	Sample	Grid
2011	1	J18
○	2	I15
	3	H14
	4	J10
	5	L10
	6	D20
	7	D19
	8	J14
	9	N11
2012	1	H17
△	2	G16
	3	C16
	4	B13
	5	E13
	6	H15
	7	I15
2013	1	I10
□	2	K9
	3	M8
	4	N6
	5	P5
	6	I11
	7	K12
	8	J14
	9	I14

Figure S1.32. Maps showing (a) the locations of Coastwide Reference Monitoring System (CRMS) and Oyster Sentinel (OS) stations closest to our sampling sites, and (b) & (c) satellite images showing the specific locations of our sample sites in each year of sampling (circles = 2011, triangles = 2012, squares = 2013). The reference table (d) lists the grid coordinates of each sample in panel (c). Satellite images are modified from Google Earth Pro version 7.1.2.2041.

Table S1.12. Table showing abundances of oysters in each sample. DA = death assemblage; LA = live assemblage.

Year	Sample	GPS coordinates	DA/LA	# specimens (≥65 mm)	# specimens (Total)
2011	1	29.306014, -89.970633	DA	18	429
			LA	3	42
2011	2	29.306258, -89.970683	DA	15	499
			LA	0	38
2011	3	29.306542, -89.971667	DA	55	331
			LA	27	63
2011	4	29.306181, -89.972878	DA	31	801
			LA	20	58
2011	5	29.305856, -89.972872	DA	42	1062
			LA	9	76
2011	6	29.307358, -89.970272	DA	9	132
			LA	7	45
2011	7	29.3073, -89.970508	DA	33	245
			LA	11	59
2011	8	29.305992, -89.971703	DA	18	556
			LA	17	25
2011	9	29.305264, -89.972436	DA	28	776
			LA	12	50
2012	1	29.306579, -89.970993	LA	5	28
2012	2	29.306799, -89.971214	LA	10	37
2012	3	29.307544, -89.971177	LA	13	62
2012	4	29.307932, -89.971873	LA	20	23
2012	5	29.307216, -89.972016	LA	2	17
2012	6	29.30653, -89.971375	LA	23	78
2012	7	29.306271, -89.971536	LA	9	59
2013	1	29.306222, -89.972861	LA	8	50
2013	2	29.305806, -89.972944	LA	7	148
2013	3	29.3055, -89.973278	LA	5	48
2013	4	29.305333, -89.973722	LA	8	45
2013	5	29.304778, -89.974056	LA	11	50
2013	6	29.306389, -89.972417	LA	12	60
2013	7	29.305889, -89.972222	LA	4	100
2013	8	29.306, -89.971722	LA	14	228
2013	9	29.306278, -89.971639	LA	15	228

Several fringing oyster beds in a small embayment on the northeast end of Grand Isle (Figs. S1.33, S1.37) were sampled in 2011–2013. The oyster beds occurred along a marsh grass edge on the northeast side of the embayment. This locality was categorized as an impact area because SCAT surveys observed heavy oiling adjacent to this location (Fig. S1.34). The locality was sampled three times for surface samples, in 2011, 2012, and 2013. Death assemblage samples were collected in 2011 (Table S1.13). Oyster right valves ≥65 mm in height were abundant in both the living and death assemblages at this locality, although abundances declined from 2011 to 2013 (Table S1.14). The average oyster heights varied between years, with 2012 being higher than the death assemblage baseline and 2011 and 2013 falling below the baseline (Fig. S1.35).

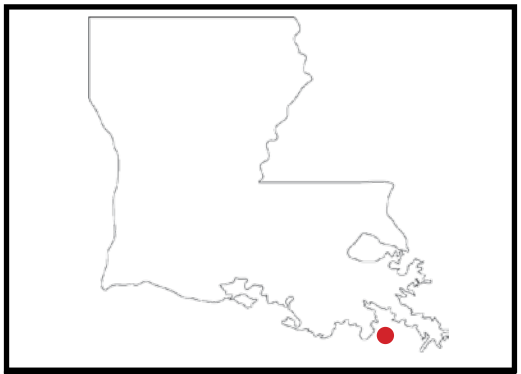

Figure S1.33. Map showing the location (red dot) of Grand Isle in Louisiana.

Environmental data from the two nearest Coastwide Reference Monitoring System (CRMS) stations to all of the Barataria Bay sampling sites (CRMS 0171: 29°19’25.68”N, 89°47’45.6”W; ~16.5 km east of nearest Grand Isle sampling sites and CRMS 0178: 29°17’15.00”N, 90°2’49.20”W; ~9.5 km northwest of nearest Grand Isle sampling sites) showed that between 2007–2013, average water temperature ± se during the summer months, May–August, was 29.0ºC ± 0.18 and 29.04ºC ± 0.17 at CRMS 0171 and 0178, respectively (Fig. S1.36). Average salinity ± se over the same interval was 14.4‰ ± 1.57 at CRMS 0171 and 16.1‰ ± 1.17 at CRMS 0178 (Fig. S1.36). Data on the prevalence of *Perkinsus marinus* (dermo disease) infection from the nearest Oyster Sentinel station to the sampling sites (Middle Hackberry Bay: 29°24’6.12”N, 90°1’48”W; ~16.5 km northwest of nearest Grand Isle sampling sites) indicated that average dermo prevalence ± se in southern Barataria Bay was 0.28 ± 0.12 between 2005 and 2013 (although no data were available for 2011 or 2012; Fig. S1.36). All environmental data

Year	DA	LA		
	2011	2011	2012	2013
Samples	6	6	6	6

Table S1.13. Table showing the year(s) Grand Isle was sampled and the number of samples collected. DA = death assemblage; LA = live assemblage.

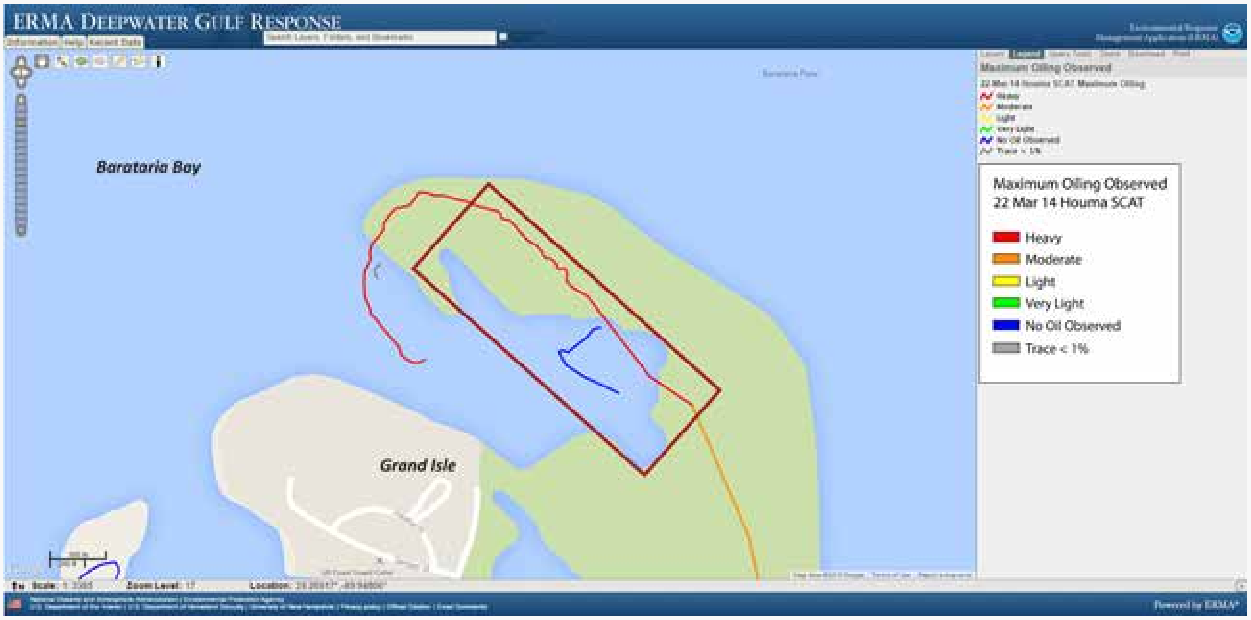

Figure S1.34. Modified screenshot from the ERMA Deepwater Gulf Response online mapping tool showing our sampling area (red box) in relation to the maximum shoreline oiling observed during Shoreline Cleanup Assessment Technique surveys. ERMA mapping tool: http://response.restoration.noaa.gov/maps-and-spatial-data/environmental-response-management-application-erma/erma-gulf-response.html (accessed 11/21/2016).

and dermo prevalence data were accessed from www.lacoast.gov/crms and www.oystersentinel.org, respectively.

Average oyster body size fell below the pre-spill baseline in 2011 and 2013, but was higher than the baseline in 2012 (Fig. S1.35), suggesting these changes were not related to the DWH oil spill. The increase in salinity in 2012 may have been beneficial for oyster growth, but would have been unlikely to counteract negative effects of the spill. There were also no trends in temperature that could have obscured the DWH oil spill’s effects on oyster body size. Likewise, dermo prevalence was below levels expected to cause oyster mortality in all years for which data were available, so could not have masked effects of the DWH oil spill (Fig. S1.36).

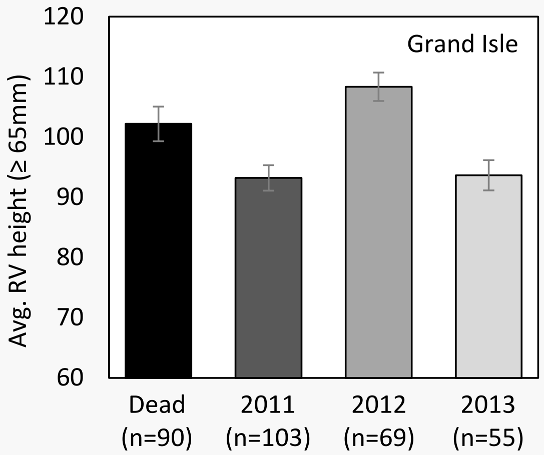

Figure S1.35. Average size of live oyster right valves (RV) ≥65 mm shell height in each year of sampling. Error bars represent the standard error of the mean.

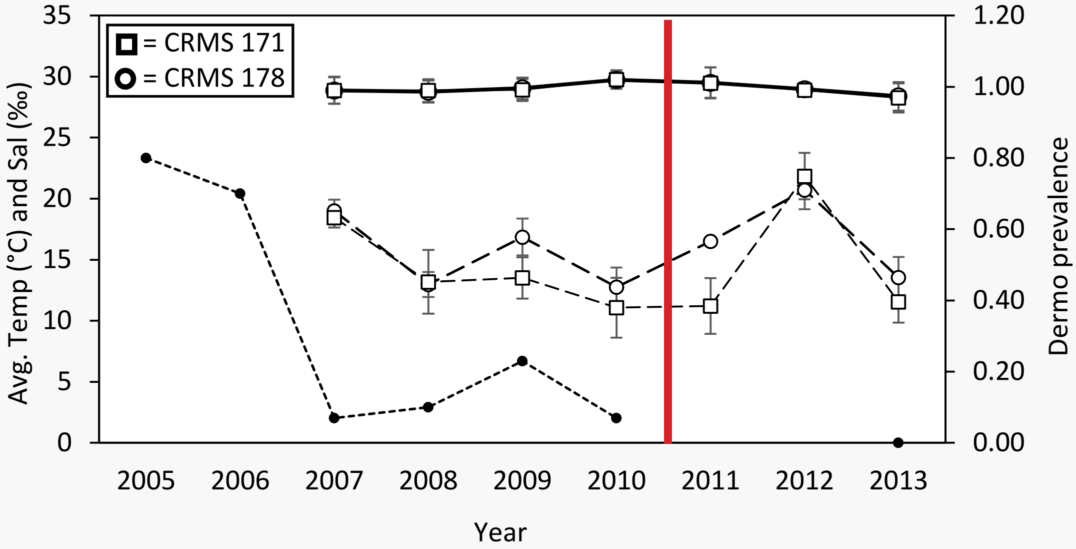

Figure S1.36. Trends in temperature (solid lines), salinity (long-dashed lines), and dermo prevalence (short-dashed line) through time at Coastwide Reference Monitoring System (CRMS; white points) and Oyster Sentinel (OS; black points) stations nearest our sampling sites. Error bars represent the standard error of the mean. Red bar indicates approximate timing of the DWH oil spill.

Data sources

Salinity & temperature data: www.lacoast.gov/crms
A) CRMS 0171 - 29°19’25.68”N, 89°47’45.6”W; ~16.5 km northeast of nearest Grand Isle sampling sites
B) CRMS 0178 - 29°17’15.00”N, 90°2’49.20”W; ~9.5 km northwest of nearest Grand Isle sampling sites

Dermo prevalence data: www.oystersentinel.org (Middle Hackberry Bay - 29°24’6.12”N, 90°1’48”W; ~16.5 km northwest of nearest Grand Isle sampling sites)

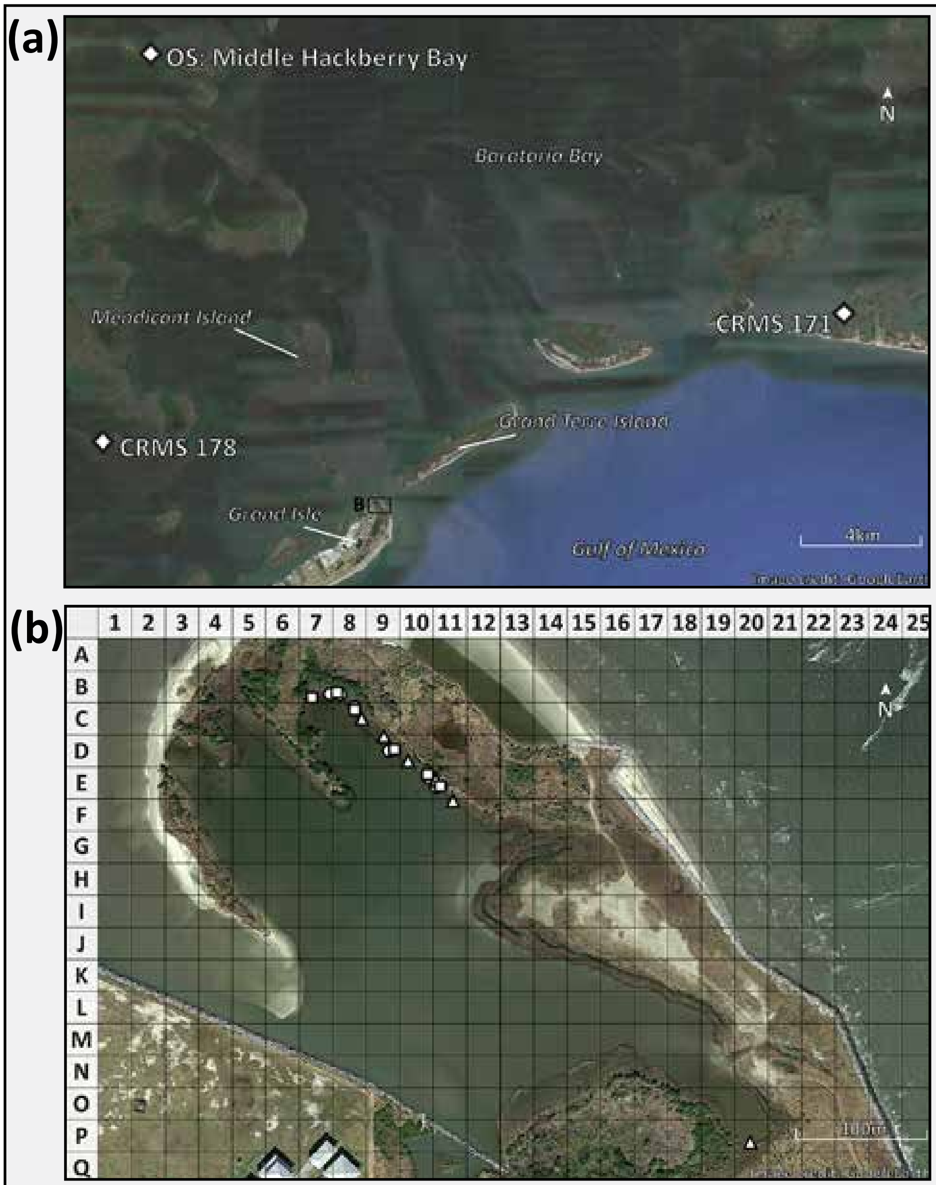

(c)

Year	Sample	Grid
2011	1	E11
○	2	E10
	3	D9
	4	C8
	5	B8
	6	B7
2012	1	P20
△	2	C8
	3	D9
	4	D10
	5	E11
	6	F11
2013	1	B7
□	2	B8
	3	C8
	4	D9
	5	E10
	6	E11

Figure S1.37. Maps showing (a) the locations of Coastwide Reference Monitoring System (CRMS) and Oyster Sentinel (OS) stations closest to our sampling sites, and (b) a gridded satellite image showing the specific locations of our sample sites in each year of sampling (circles = 2011, triangles = 2012, squares = 2013). The reference table (c) lists the grid coordinates of each sample in panel (b). Satellite images are modified from Google Earth Pro version 7.1.2.2041.

Table S1.14. Table showing abundances of oysters in each sample. DA = death assemblage; LA = live assemblage.

Year	Sample	GPS coordinates	DA/LA	# specimens (≥65 mm)	# specimens (Total)
2011	1	29.268428, -89.953969	DA	19	308
			LA	16	23
2011	2	29.268486, -89.954019	DA	9	166
			LA	16	20
2011	3	29.268672, -89.954317	DA	10	151
			LA	28	38
2011	4	29.268961, -89.954581	DA	19	140
			LA	25	43
2011	5	29.269047, -89.954719	DA	22	203
			LA	7	18
2011	6	29.26905, -89.954764	DA	11	119
			LA	11	18
2012	1	29.265986, -89.95156	LA	2	18
2012	2	29.268884, -89.954534	LA	8	22
2012	3	29.268758, -89.954355	LA	15	29
2012	4	29.268594, -89.954171	LA	25	33
2012	5	29.268454, -89.953971	LA	5	36
2012	6	29.26833, -89.95383	LA	14	128
2013	1	29.269028, -89.954917	LA	13	85
2013	2	29.269056, -89.954722	LA	11	51
2013	3	29.268944, -89.954583	LA	5	39
2013	4	29.268667, -89.954278	LA	15	48
2013	5	29.2685, -89.954028	LA	7	46
2013	6	29.268417, -89.953944	LA	4	43

8. Grand Terre Canal (29°16’36.85”N, 89°56’31.70”W) Treatment: Impact

Several oyster beds in the vicinity of a small canal cutting into the northwestern end of Grand Terre Island (Figs. S1.38, S1.40) were sampled in 2011–2013. The oyster beds sampled were fringing beds that occurred in pools and channels along the marsh on the eastern side of the canal and along the shore of Barataria Bay a short distance west of a channel connecting the canal with the bay (Fig. S1.40). This locality was categorized as an impact area because SCAT surveys observed heavy oiling adjacent to these locations. The locality was sampled three times for surface samples, in 2011, 2012, and 2013. Death assemblage samples were collected in 2011 (Table S1.15). Oyster right valves ≥65 mm in height declined in abundance between 2011 and 2013 (Table S1.16), but there was a slight increase in the average heights of the oysters in 2012 that continued into 2013 (Fig. S1.39). Grand Terre Canal was the only site with a precipitous decline in abundances. The temporal pattern in average body sizes at this locality is similar to Mendicant Island, where average body sizes declined from 2011 to 2012, but not from 2012 to 2013 (Fig. S1.29). Further, the decline in abundances of large oysters is in contrast to the total abundances of oysters of all sizes, which did not decrease from 2011 to 2013 (Table S1.16).

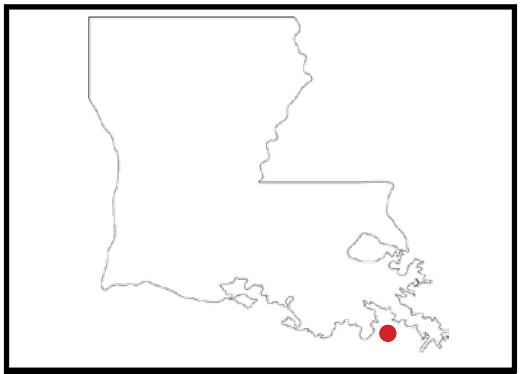

Figure S1.38. Map showing the location (red dot) of Grand Terre Island in Louisiana.

Year	DA	LA		
	2011	2011	2012	2013
Samples	9	9	9	9

Table S1.15. Table showing the year(s) Grand Terre Canal was sampled and the number of samples collected. DA = death assemblage; LA = live assemblage.

Environmental data from the two nearest Coastwide Reference Monitoring System (CRMS) stations to all of the Barataria Bay sampling sites (CRMS 0171: 29°19’25.68”N, 89°47’45.6”W; ~15 km northeast of nearest Grand Terre Canal sampling sites and CRMS 0178: 29°17’15.00”N, 90°2’49.20”W; ~10.25 km northwest of nearest Grand Terre Canal sampling sites) showed that between 2007–2013, average water temperature ± se during the summer months, May–August, was 29.0°C ± 0.18 and 29.04°C ± 0.17 at CRMS 0171 and 0178, respectively (Fig. S1.41). Average salinity ± se over the same interval was 14.4‰ ± 1.57 at CRMS 0171 and 16.1‰ ± 1.17 at

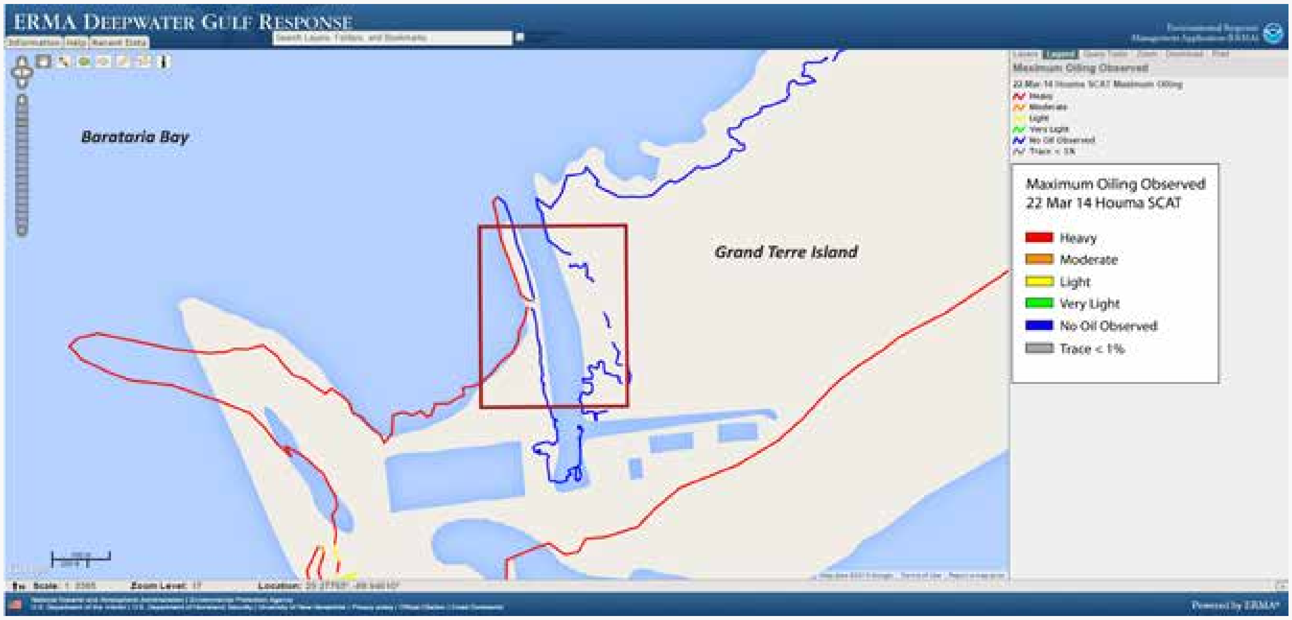

Figure S1.39. Modified screenshot from the ERMA Deepwater Gulf Response online mapping tool showing our sampling area (red box) in relation to the maximum shoreline oiling observed during Shoreline Cleanup Assessment Technique surveys. ERMA mapping tool: http://response.restoration.noaa.gov/maps-and-spatial-data/environmental-response-management-application-erma/erma-gulf-response.html (accessed 11/21/2016).

8. Grand Terre Canal (29°16’36.85”N, 89°56’31.70”W) Treatment: Impact

CRMS 0178 (Fig. S1.41). Data on the prevalence of *Perkinsus marinus* (dermo disease) infection from the nearest Oyster Sentinel station to the sampling sites (Middle Hackberry Bay: 29°24’6.12”N, 90°1’48”W; ~16.5 km northwest of nearest Grand Isle sampling locations) indicated that average dermo prevalence ± se in southern Barataria Bay was 0.28 ± 0.12 between 2005 and 2013 (although no data were available for 2011 or 2012; Fig. S1.41). All environmental data and dermo prevalence data were accessed from www.lacoast.gov/crms and www.oystersentinel.org, respectively (Fig. S1.41).

Average oyster body size increased in 2012 and 2013 above the pre-spill baseline (Fig. S1.40), opposite the expected pattern if the DWH oil spill had negatively affected oyster growth. The increase in salinity in 2012 may have been beneficial for oyster growth, but would have been unlikely to counteract negative effects of the spill. There were no trends in temperature that could have obscured the DWH oil spill’s effects on oyster body size. Likewise, dermo prevalence was below levels expected to cause oyster mortality in all years for which data were available, so could not have masked effects of the DWH oil spill (Fig. S1.41).

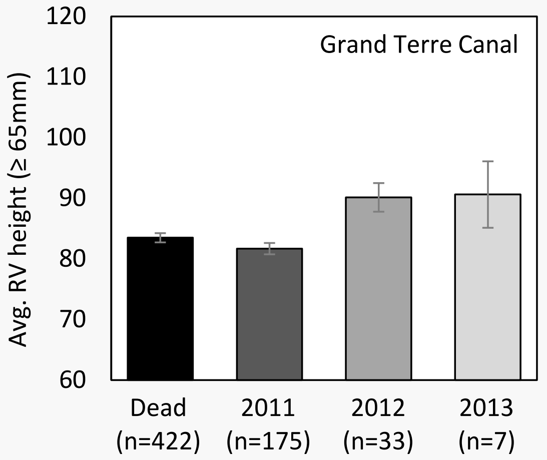

Figure S1.40. Average size of live oyster right valves (RV) ≥65 mm shell height in each year of sampling. Error bars represent the standard error of the mean.

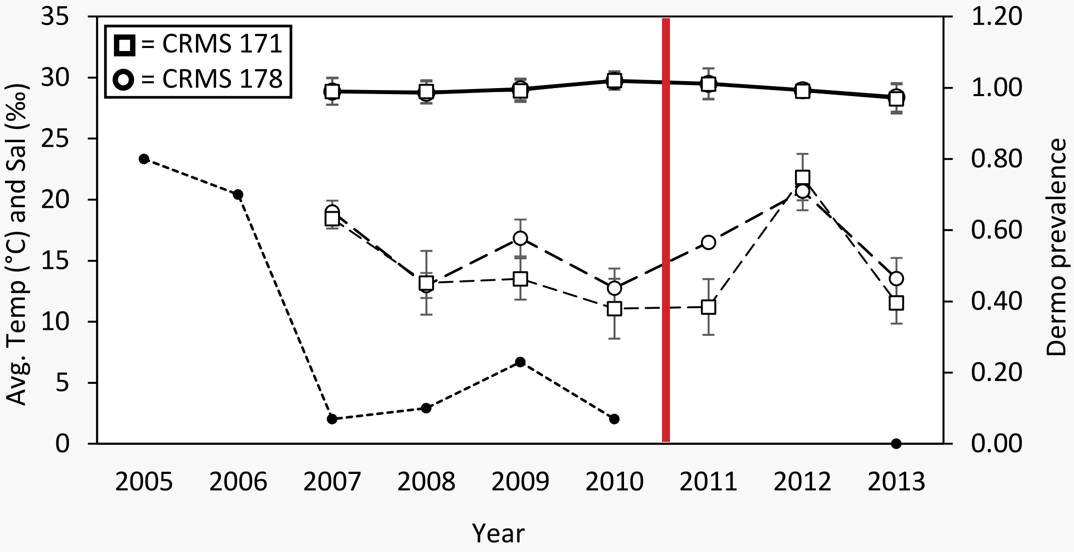

Figure S1.41. Trends in temperature (solid lines), salinity (long-dashed lines), and dermo prevalence (short-dashed line) through time at Coastwide Reference Monitoring System (CRMS; white points) and Oyster Sentinel (OS; black points) stations nearest our sampling sites. Error bars represent the standard error of the mean. Red bar indicates approximate timing of the DWH oil spill.

Data sources

Salinity & temperature data: www.lacoast.gov/crms
A) CRMS 0171 - 29°19’25.68”N, 89°47’45.6”W; ~15 km northeast of nearest Grand Terre Canal sampling sites
B) CRMS 0178 - 29°17’15.00”N, 90°2’49.20”W; ~10.25 km northwest of nearest Grand Terre Canal sampling sites

Dermo prevalence data: www.oystersentinel.org (Middle Hackberry Bay - 29°24’6.12”N, 90°1’48”W; ~16 km northwest of nearest Grand Terre Canal sampling sites)

(a) (b) (c)

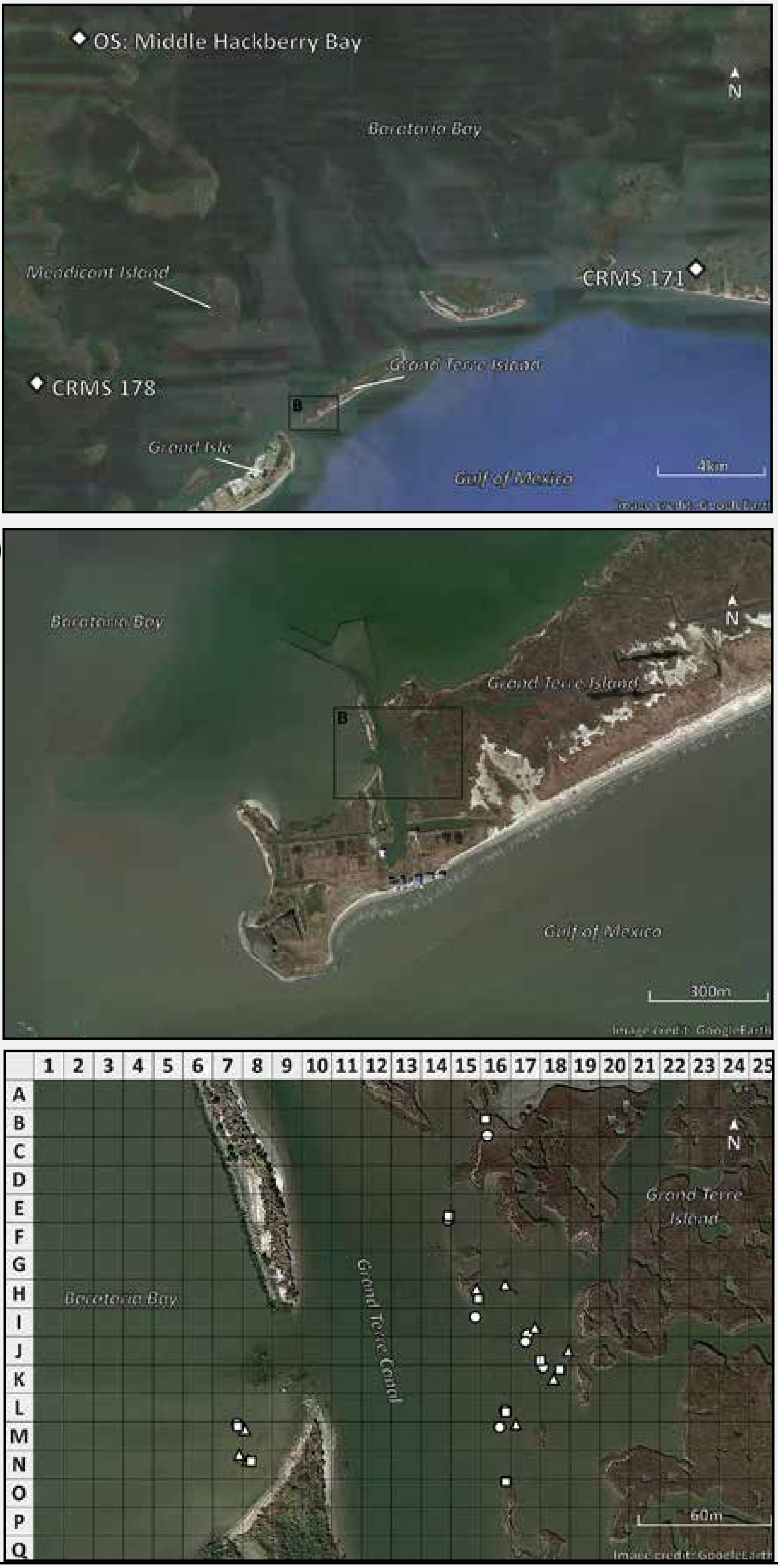

(d)

Year	Sample	Grid
2011 ○	1	J17
	2	I17
	3	K18
	4	L16
	5	M16
	6	E14
	7	I15
	8	B16
	9	M7
2012 △	1	H15
	2	H16
	3	I17
	4	J18
	5	K18
	6	M17
	7	M8
	8	N7
	9	N8
2013 □	1	H15
	2	E14
	3	B16
	4	J17
	5	K18
	6	L16
	7	O16
	8	M7
	9	N8

Figure S1.42. Maps showing (a) the locations of Coastwide Reference Monitoring System (CRMS) and Oyster Sentinel (OS) stations closest to our sampling sites, and (b) & (c) satellite images showing the specific locations of our sample sites in each year of sampling (circles = 2011, triangles = 2012, squares = 2013). The reference table (d) lists the grid coordinates of each sample in panel (c). Satellite images are modified from Google Earth Pro version 7.1.2.2041.

8. Grand Terre Canal (29°16’36.85”N, 89°56’31.70”W) Treatment: Impact

Table S1.16. Table showing abundances of oysters in each sample. DA = death assemblage; LA = live assemblage.

Year	Sample	GPS coordinates	DA/LA	# specimens (≥65 mm)	# specimens (Total)
2011	1	29.277078, -89.941761	DA	74	319
			LA	32	49
2011	2	29.277103, -89.941753	DA	67	268
			LA	23	39
2011	3	29.276978, -89.941686	DA	18	108
			LA	14	24
2011	4	29.276806, -89.941864	DA	10	793
			LA	5	29
2011	5	29.276744, -89.941886	DA	58	726
			LA	4	31
2011	6	29.277569, -89.942106	DA	46	465
			LA	33	76
2011	7	29.277181, -89.941989	DA	39	304
			LA	20	55
2011	8	29.277897, -89.941928	DA	80	333
			LA	25	37
2011	9	29.276764, -89.943078	DA	30	628
			LA	19	66
2012	1	29.277283, -89.941986	LA	0	8
2012	2	29.277304, -89.941855	LA	4	14
2012	3	29.277139, -89.941726	LA	4	15
2012	4	29.277046, -89.941571	LA	5	14
2012	5	29.276933, -89.941638	LA	4	12
2012	6	29.276754, -89.941807	LA	1	24
2012	7	29.276742, -89.943046	LA	8	41
2012	8	29.276636, -89.943073	LA	0	22
2012	9	29.276617, -89.943048	LA	7	27
2013	1	29.27725, -89.941972	LA	0	65
2013	2	29.277583, -89.942111	LA	0	29
2013	3	29.277972, -89.941944	LA	0	42
2013	4	29.277, -89.941694	LA	0	25
2013	5	29.276972, -89.941611	LA	1	87
2013	6	29.276806, -89.941861	LA	0	87
2013	7	29.276528, -89.941861	LA	1	105
2013	8	29.27675, -89.943083	LA	3	43
2013	9	29.27661, -89.94304	LA	2	11
