# Supplementary material for: Geohistorical records indicate no impact of the Deepwater Horizon oil spill on oyster body size
Source: R Soc Open Sci. 2016 Nov 30;3(11):160763. doi: 10.1098/rsos.160763 (PMC5180161; doi:10.1098/rsos.160763)
Supplement: Electronic supplementary material 2: Analysis of oyster body size distributions [file rsos160763supp2.pdf]

Geohistorical records indicate no impact of the Deepwater Horizon oil spill on oyster body size

Gregory P. Dietl, Stephen R. Durham

Electronic supplementary material S2: Analysis of oyster body-size distributions

In order to ensure that our BACI analysis of average oyster body sizes before and after the Deepwater Horizon (DWH) oil spill was not missing important variation in the distribution of oyster sizes, we compared the distribution of body sizes of oysters ≥65 mm shell height at control and impact locations in each year using Komolgorov-Smirnov tests (table S2.1). All K-S tests were not significant, suggesting that the distribution of body sizes among large oysters was similar in all collection year categories. Had the DWH oil spill affected the distribution of body sizes, we expected that growth would decrease and/or mortality would increase, likely shifting the distribution of body sizes at impacted locations towards smaller sizes, but this was not the case. Further, when the BACI analysis we conducted using average oyster body size was repeated using median body size as the response, the results were very similar (BACI contrast estimate = 2.0 ± 3.14 s.e., $p = 0.532$; figure S2.1; Table S2.2), suggesting our conclusions based on the analysis of means are robust.

Table S2.1. Results of Komolgorov-Smirnov tests comparing the distribution of oyster body sizes between control and impact groups in each time bin.

	count				
time bin	control	impact	*KS*	*D*	*p*
Dead	1000	864	0.03	0.06	0.068
2011	301	384	0.03	0.05	0.796
2012	226	184	0.04	0.09	0.411
2013	392	285	0.04	0.09	0.139

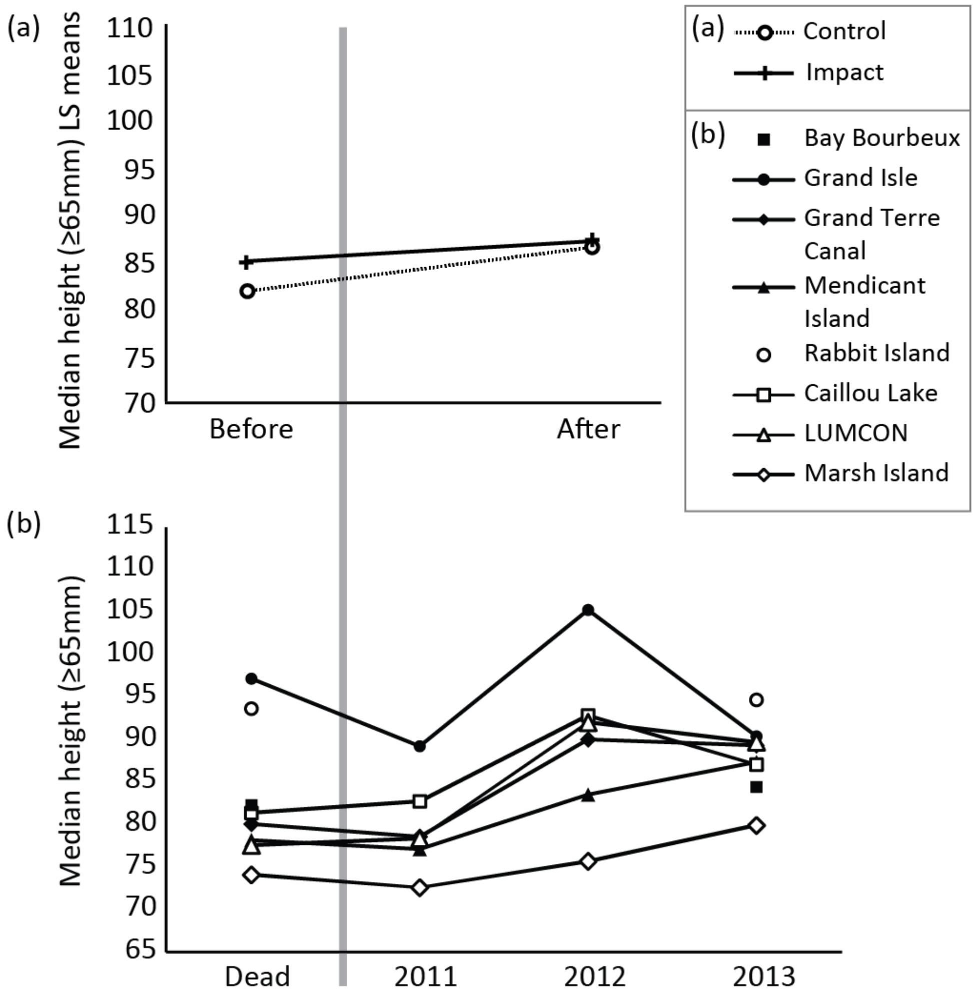

Fig. S2.1. (a) Plot showing the least squares means of the interaction effect from the linear mixed model comparing treatment, time, and treatment x time, and including locality and year as random effects. This plot shows a visual representation of the BACI contrast (i.e., the difference in the differences of median body sizes between control and impact localities before and after the spill). The lines for impact and control treatments are nearly parallel, indicating a lack of treatment x time interaction. (b) Plot showing trends in median heights of oyster right valves (≥65 mm) from eight localities in Louisiana that either received moderate/high levels of maximum oiling (black shapes; total *n*=1,717) or had no oil observed in the vicinity (white shapes; total *n*=1,919). The vertical grey line indicates data from oysters that lived prior to the DWH spill (Before/Dead) and those that lived through or recruited following the spill (After/2011–2013).

Table S2.2. Fixed effects results of a linear mixed model assessing the impact of treatment, time, and their interaction on the median body size of oysters ≥65 mm in shell height.

fixed effect	*F*	*p*
Treatment*	$F_{1,\ 6.08} = 0.12$	0.738
Time†	$F_{1,\ 1.94} = 0.34$	0.622
Treatment x Time	$F_{1,\ 16.11} = 0.41$	0.532

*Control or impact

†Before or after the spill
